# Charactering tumor microenvironment reveals stromal‐related transcription factors promote tumor carcinogenesis in gastric cancer

**DOI:** 10.1002/cam4.3133

**Published:** 2020-05-28

**Authors:** Haining Liu, Shujuan Ni, Hanbo Wang, Qiongyan Zhang, Weiwei Weng

**Affiliations:** ^1^ Department of Gastroenterology and Hepatology Zhongshan Hospital Fudan University Shanghai P.R. China; ^2^ Department of Oncology Shanghai Medical College Fudan University Shanghai China; ^3^ Department of Pathology Fudan University Shanghai Cancer Center Shanghai China; ^4^ Department of Pathology Zhongshan Hospital Fudan University Shanghai China; ^5^ Jining Medical University Jining China

**Keywords:** CDH11, gastric cancer, HEYL, transcriptional factors, tumor microenvironment

## Abstract

Transcription factors represent the crucial role of controlling gene transcription in cancer development and progression. However, their functions in gastric cancer have not been thoroughly characterized. For this study, we comprehensively evaluated the correlation between infiltration patterns of tumor microenvironment (TME) cells and TFs expression in the cohort of stomach adenocarcinoma (STAD) from TCGA database. We integrally explored differential expression panel and prognostic value of candidate TFs in TCGA‐STAD cohort. Notably, we found a key transcription factor named HEYL, which its expression level was correlated with stromal component transformation of TME. HEYL was regularly high expressed in gastric cancer and correlated with patients’ poor prognosis. Knockdown of HEYL prominently abrogated the tendency of cell proliferation, migration, and progression in gastric cancer. Consistently, overexpression of HEYL strikingly accelerated the gastric carcinoma development through activating oncogenic signaling pathways and transcriptional activation of cadherin 11 (CDH11). Our findings not only identified the close relationship between TFs and TME phenotype, but also emphasized the crucial importance of TFs, especially HEYL, which could be identified as a candidate biomarker to evaluate prognostic risk and therapeutic effect in gastric cancer.

## Graphical abstract

Our data highlighted that TME‐related TFs, especially HEYL, may represent a potentially promising target of diagnosis and clinical interventions for gastric cancer patients.

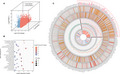

## 1 INTRODUCTION

Gastric cancer (GC) is known as a gastrointestinal malignance with dismal clinical outcome and shows increasing incidence and mortality worldwide.^1^ Even though the medical applications and clinical managements have developed such as immunotherapy and neoadjuvant therapy, the curative effect and 5‐year survival rate of gastric cancer remains to be improved.^2^ Affirmatively, the developing cancer genomic method has shown the important technology used in clinical and fundamental institutions to investigate the novel diagnostic and therapeutic target in gastric cancer. Recent researches have indicated the significance between tumor‐related constituents and signaling pathways in tumor microenvironment and malignant cells, indicating the exploration of intracellular relationship is more significant at single cell perspective.^3^, ^4^, ^5^

A large amount of works suggested the vital roles of TME formation or transition in tumor progression and therapeutic response.^6^, ^7^ Indeed, the diverse stromal components such as fibroblasts and mesenchymal stromal cells could serve as the mediators for tumor stromal cells intensive communications and ultimately coordinate the construction of tumorigenic microenvironment.^8^

Transcriptional factors (TFs) are crucial regulatory factors in orchestrating gene expression in the duration of cancer development. Several investigations uncovered TFs conduct potential functions in remodeling TME cells transformation and control cancer cells proliferation and migration.^9^, ^10^ Meanwhile, studies have also focused on understanding biological functions and potential regulated networks of TFs in stromal components of GC, especially for cancer‐associated fibroblasts (CAFs), and tumor‐associated mesenchymal stromal cells (TA‐MSCs).^10^, ^11^, ^12^ To date, the comprehensive investigation among the TFs landscape and TME components with their molecular functions of carcinogenesis in gastric cancer has not yet been completely elucidated.

In our current research, we systematically curated the TFs prognostic risks and correlated their characteristics on GC‐TME heterogeneity by investigating the TCGA‐STAD transcriptional profiles. We found that high‐risk TFs were associated with activation of cancer‐related signaling and TME remodeling, especially in mesenchymal component accumulation and stromal cell infiltrating. Moreover, we identified a key transcriptional factor HEYL that significantly promotes stromal component accumulation and serves as an independent prognostic biomarker in GC. Highly expressed HEYL was frequently observed in STAD malignant tissues and associated with patient's dismal survival. Moreover, we found HEYL enhances gastric carcinogenesis through activating oncogenic signaling pathways and regulating CDH11 expression under DNA level in GC cells. In summary, we precisely demonstrated a quantified TME infiltration pattern remodeling by TFs and found HEYL could represent a potentially prognostic and therapeutic target in gastric cancer.

## 2 MATERIALS AND METHODS

### 2.1 Human specimen and survival information

The 117 paired tumor and paracancerous tissues were acquired from the Zhongshan Hospital, Fudan University (Shanghai, China) in the duration between September 2015 and August 2019. Informed consent was signed by all patients. All the 117 patients with gastric cancer have OS information, among which 107 patients include DFS follow‐up.

### 2.2 Statistical analysis

The different expression results of candidate transcription factors in TCGA‐STAD cohort was conducted by moderate Student's *t* test using R package *limma*. The quantitative variables were preferentially analyzed by Shapiro‐Wilk normality test to validate their normality, then the statistical results for normally distributed data were analyzed by Student's *t* test, the non‐normally distributed variables were further estimated by Mann‐Whitney *U* tests. We used the Kaplan‐Meier analysis to construct OS and DFS cumulative curves for the binomial classes, and the log‐rank test was conducted to evaluate the statistical significance between two cumulative plots. The hazard ratios of univariate and multivariate analysis were generated from Cox proportional hazard regression analysis.

The comparison of diagnosis performance between HEYL and traditional gastrointestinal biomarker CA19‐9 was analyzed by receiver operating characteristic curve analysis. The statistical differences between two ROC plots and the AUC with 95% confidence intervals were computed by DeLong test. The R platform (V3.5.2, https://www.r‐project.org/) was implemented to conduct all statistical analyses. All statistical analyses were executed by two‐sided test and the test results shown *P*‐value < .05 were considered as statistically significant. The R packages “Limma”, “pROC”, “survival”, “clusterProfiler”, and “pheatmap” were used in this study**.**

Others detailed materials and methods are presented in the Supplementary Material

## 3 RESULTS

### 3.1 The differentially expressed transcription factors with prognostic significance in TCGA‐STAD cohort

**Figure 1:**
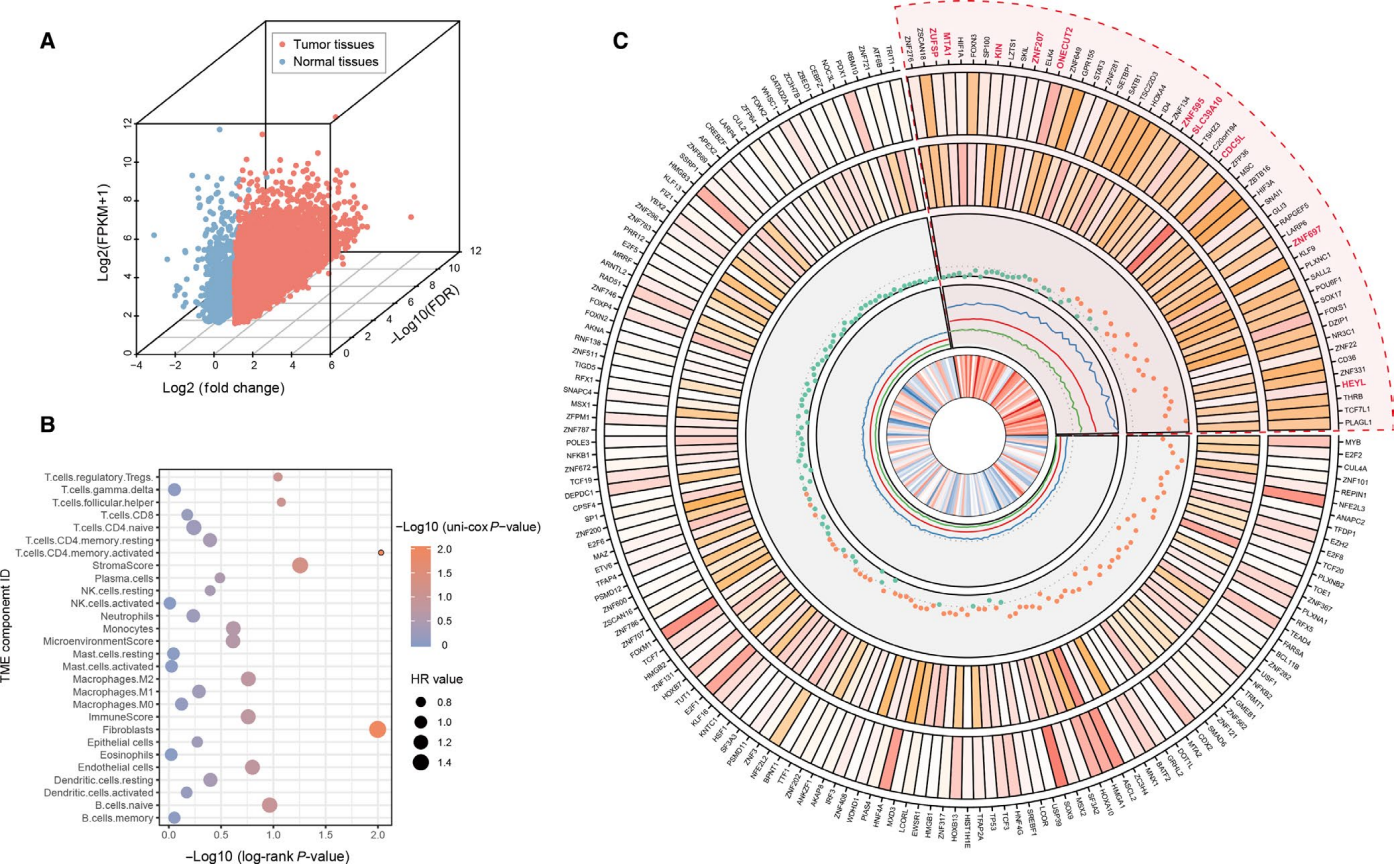
Differentially expressed transcription factors with prognostic significance in TCGA‐STAD cohort. (A) Three‐dimensional scatter plot of dysregulated TF genes in gastric cancer. (B) The circos drawing generated by all candidate TFs with their statistical results from TCGA‐STAD cohort. The figure constructs by five panel: i) the first panel indicates the log2 transfer average expression level of TFs in tumor samples; ii) the second panel shows the log2 transferred mean fold‐change of selected TFs compared by tumor and normal tissues; iii) the third panel shows the *P*‐value (–log10 transferred) of log‐rank test; iv) the fourth panel demonstrates the hazard ratio of univariate‐cox test of each TF, the red, blue, and green line refer to the HR and its upper and lower 95% CI, respectively; v) the last panel refers the correlation coefficients (R) generated from the results by comparing tumor stage and TFs expression level. The higher the value, the darker crimson color highlighted in the cell. (C) The prognosis evaluation of tumor microenvironment cell types. The size of each bubble represents the HR value of each TME fraction type, and the OS results calculated used the formula –log 10 (Log‐rank test *P*‐value)

We first analyzed the differential expression pattern of 1935 TF genes in GC tissue. In TCGA‐STAD cohort, we selected all 27 paired tissues (tumor and normal tissue) as an expression profile to conduct different expressed analysis for each TF gene. The results indicated that 419 TFs were upregulated in gastric cancer compared with paracarcinoma samples, whereas 64 genes were downexpressed in tumor (Figure 1A; Table S1).

We then explored the clinical significance of differentially expressed TFs, an overall survival analysis was executed to estimate the association between these different expressed TFs and patient's prognostic outcome. The TFs transcriptional profiles of 370 GC samples (gene level, quantified by FPKM) and clinical information in TCGA‐STAD cohort were used for OS analysis. The tumor samples were grouped binomially by the optimal cutoff of all TFs, the OS curves were generated by Kaplan‐Meier plot and differential analysis between each two OS cumulative plot was tested by log‐rank test (see Materials and Methods). As a result, 30 downregulated TF and 159 upregulated TF were associated with patients’ over‐survival (*P* < .05; Figure 1B). Among these candidate genes, 59 TFs were shown as high risk for patients’ prognosis (hazard ratio >1, Figure 1B). Consistently, we systematically demonstrated the expression level, fold‐change, prognostic hazard ratio, and tumor stage correlation of dysregulated TFs in an apparent layout, and these prognosis‐associated TFs were selected for subsequent analysis to explore their biological functions on TME (Figure 1B).

### 3.2 The relationship between prognostic TFs and the landscape of TME in gastric cancer

To further understand and characterize the biological functions and clinical implications of selected TFs inside tumor microenvironment, we constructed the TME cells infiltrating pattern by two algorithms (see Data S1) and systematically evaluated their effects on patient's overall survival from public TCGA‐STAD database (Figure 1C, Table S2). Two main TME cells infiltration subtypes (fibroblasts and activated memory CD4 T‐cell) were correlated with prognostic evaluation in tumor tissues (*P‐*value < 0.05, log‐rank test) and one of the stromal‐related component (fibroblasts) serves as a risk factor for patient's overall survival (hazard ratio >1.53, *P* < .05; Figure 1C, Table S2).

**Figure 2:**
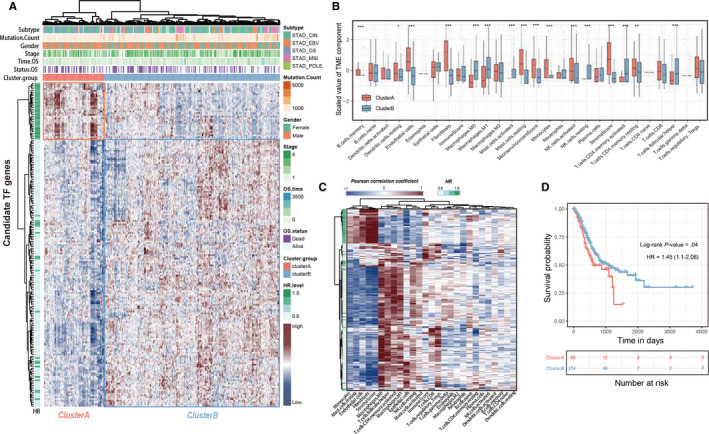
The relationship between prognostic TFs and the landscape of TME in gastric cancer. (A) Unsupervised analysis and hierarchical clustering of candidate TFs based on expression profile derived from TCGA‐STAD cohort to classify patients into two groups (indicated in a red and blue box). (B) The boxplot of TME component in two TME clusters. Within each group, the scattered dots represent scaled TME fraction expression values. The thick line represents the median value. The bottom and top of the boxes are the 25th and 75th percentiles (interquartile range). The whiskers encompass 1.5 times the interquartile range. The statistical difference of two clusters were compared through the Mann‐Whitney *U* test. (C) The hierarchical clustering heatmap represents the correlation coefficient between selected TFs and tumor microenvironment fractions. (D) Kaplan‐Meier curves for overall survival of TCGA‐STAD 370 patients with TF‐based classed. The number of patients in cluster A and B phenotypes are n = 96 and n = 274, respectively. The statistical difference between two survival curves was tested by log‐rank test. *P < .05; **P < .01; ***P < .001

We further investigated whether the candidate prognostic TFs participant in the biological processes of TME formation and reconstruction in gastric cancer. We received the selected TF genes expression profile in TCGA‐STAD cohort and stratified the total 370 samples into two groups (cluster A and cluster B) by hierarchical agglomerative clustering (Ward's linkage, Figure 2A). Most of the risk TFs were distributed in cluster A whereas the favorable factor TFs were fully recorded in cluster B. Meanwhile, the statistical differences of infiltrating TME cells among two main clustered phenotypes were detected by Mann‐Whitney *U* test (Figure 2B). We then estimated the association between the above‐mentioned TFs and infiltration patterns of TME cells, the results represented the stromal related component (endothelial cell, fibroblast and stromal cell) significantly correlated with risk‐factor‐TFs, whereas the favorable TFs showed high association with infiltration of immune faction (Figure 2C). Notably, the results of overall survival analysis shown the patients in TME cluster A (96 patients, stromal‐correlated cluster) correlated with dismal prognosis compared with TME cluster B (274 patients, immune‐correlated cluster; *P* < .05, log‐rank test; Figure 2D). These results indicated that the expression profile of prognosis‐related TFs was associated with different infiltrating pattern of tumor microenvironment components (stromal and immune cells) and by separated patients into two groups with overall survival distinction.

### 3.3 Identification of the stromal‐correlated signatures and functional annotations

We further explored whether the candidate risk TFs regulate downstream targets and then perform underlying biological function in stromal transformation of TME progression. We identified 2055 differential expressed genes (DEGs) which upregulated in TME cluster A group (FDR <0.05, fold‐change >1.25) by *limma* package (see Materials and Methods) and these representative DEGs were selected to perform enrichment analysis and function annotation across TCGA‐STAD cohort.

**Figure 3:**
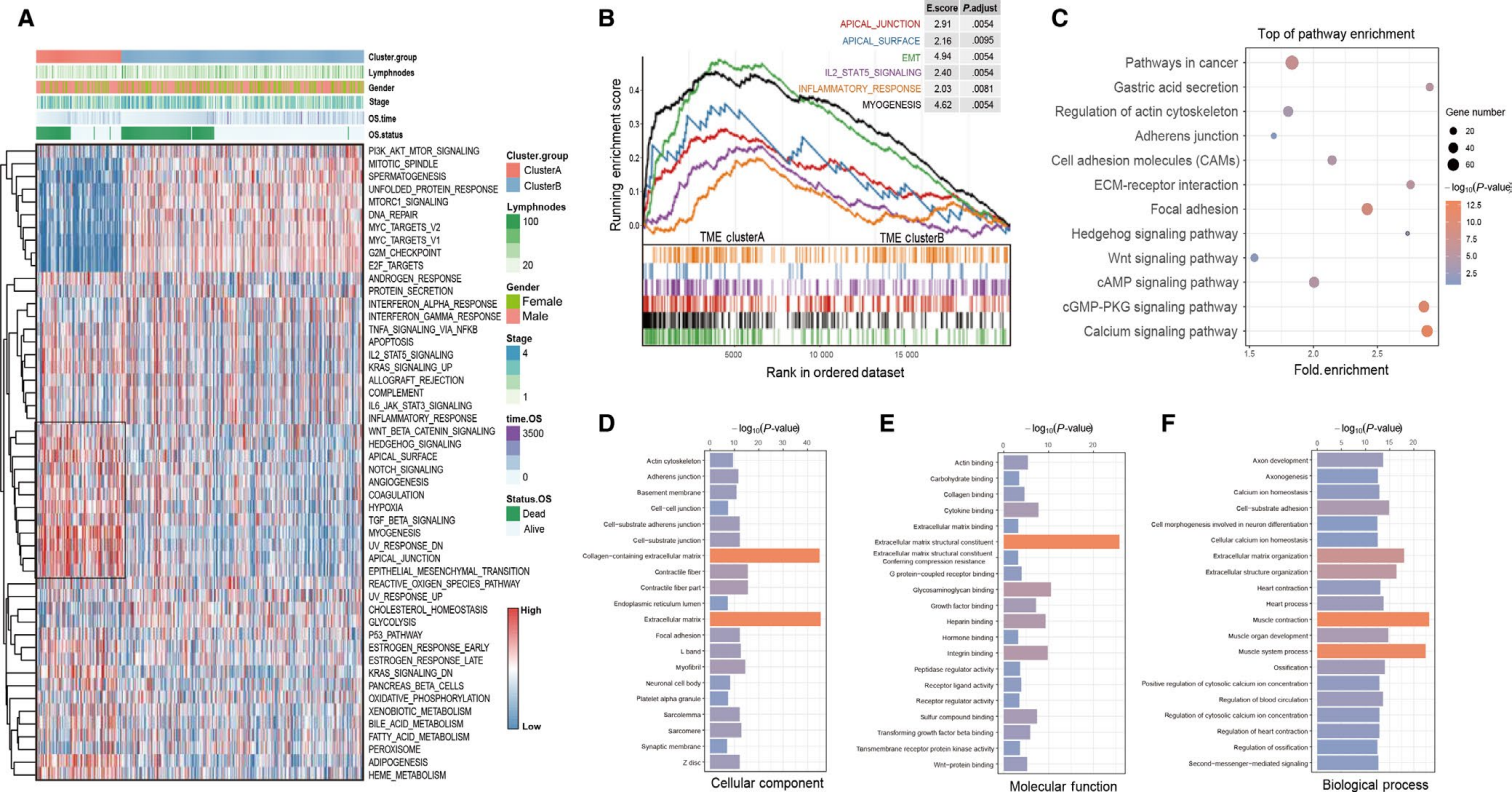
Identification of the stromal correlated signatures and functional annotations. (A) The heatmap with unsupervised analysis and hierarchical clustering shows the enrichment score of cancer hallmark gene sets enrich in cluster A/B based on the ssGSEA method for TCGA‐STAD cohort. (B) Gene set enrichment analysis (GSEA) of hallmark gene sets downloaded from MSigDB database. All transcripts were ranked by log2 (fold change) between gene clusters A and B. Each run was performed with 1,000 permutations. Enrichment results with significant associations in TME cluster A were shown on enrich plot curve. (C) KEGG pathway analysis performed by the DAVID platform for upregulated DEGs in cluster A. The KEGG pathway with *P*‐value < .05 was shown in bubble plot. (D‐F) Gene Ontology (GO) enrichment analysis of the cluster A relevant signature genes demonstrated by cell component, molecular function, and biological process module. The x‐axis indicates the *P*‐value of signatures within each GO term

We first used the single sample GSEA algorithm to depict the enrichment landscape of cancer hallmark gene signatures in total 370 TCGA‐STAD samples, as well as enrichment scores generated from each sample were fully clustered by hierarchical clustering method (Ward's linkage). The results indicated that the TGF‐β, Wnt/beta‐catenin pathway, apical junction, and epithelial‐mesenchymal transition (EMT) signatures were fully enriched in TME cluster A. Meanwhile, the subtype group of TME cluster B acquired the high enrichment level of PI3K‐AKT‐mTOR signaling, DNA repair, G2M checkpoint, and E2F targets signatures (Figure 3A). Meanwhile, we used the Gene Set Enrichment Analysis (GSEA) to identify the enrichment of cancer hallmark pathways with extract statistical results (Figure 3B).

Moreover, the results of KEGG pathway analysis showed that the gene signatures upregulated in TME cluster A were enriched in typical pathways involved in tumor development or progression such as pathways in cancer, Wnt/beta‐catenin signaling pathway, cell adhesion molecules, and adherents junctions (Figure 3C). Gene ontology enrichment analysis showed the cell component, molecular function, and biological process module referred to TME cluster A were highly associated with cellular membrane remodeling, cytoskeletal rearrangement, and the receptor activation of cancer‐related pathway (Figure 3D‐F). These functional annotation results implied the underlying biological mechanism mediated by TFs in TME elements transition and explored their downstream signaling pathways in cancer development and progression.

### 3.4 HEYL serves as a promising diagnostic biomarker and predicts prognosis in gastric cancer

**Figure 4:**
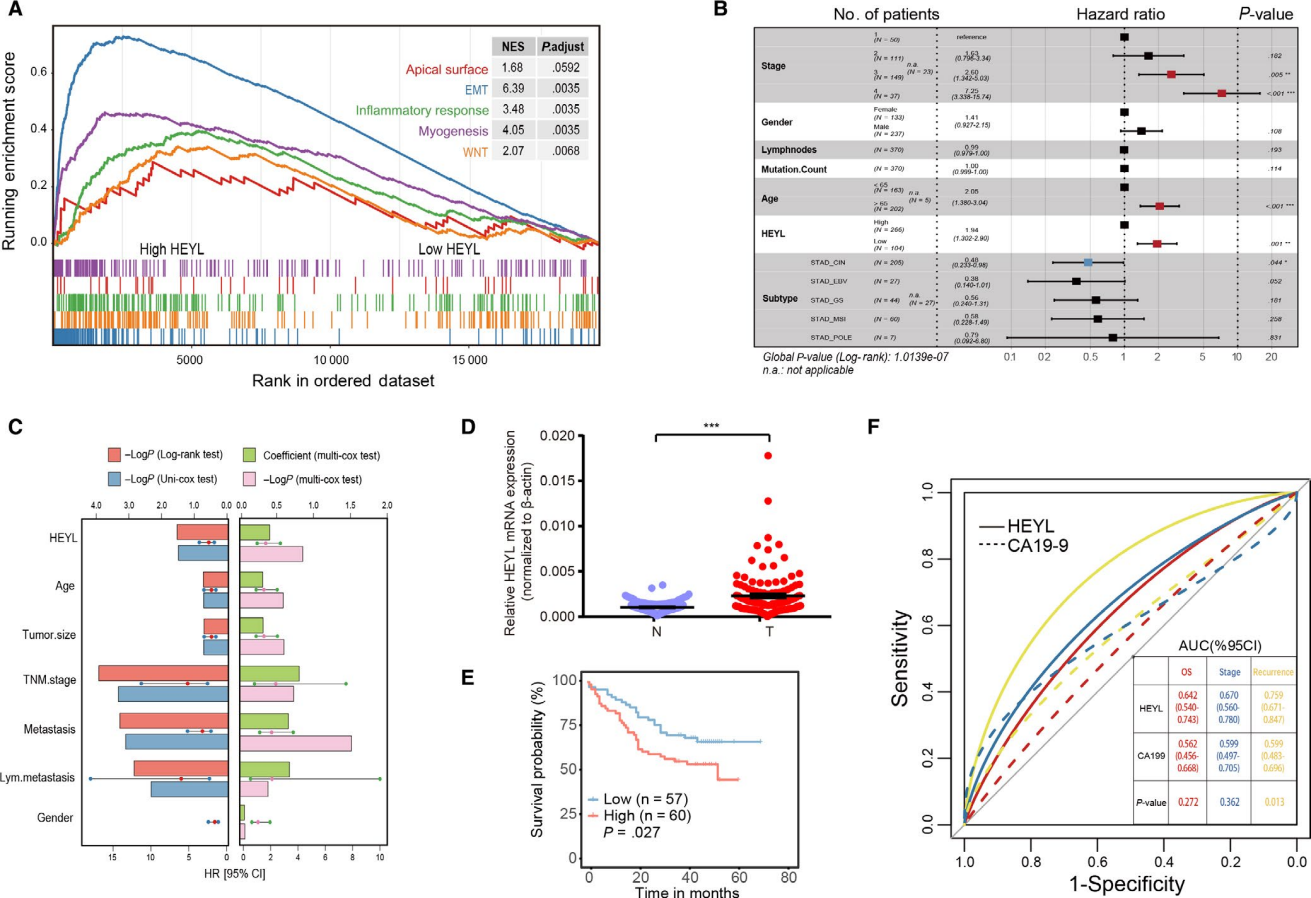
HEYL serves as a promising diagnostic biomarker and predicts prognosis in gastric cancer. (A) The GSEA results indicate the HEYL upregulated sample show significantly enriched in EMT pathway compare to HEYL downregulated group. (B) The table of multivariate analyses using the Cox proportional hazard regression model comparing HEYL expression values with other factors (age, gender, tumor stage, lymph nodes, and mutation burden and tumor subtype) as covariates. (C) The results of uni‐ and multivariate analyses using the Cox proportional hazard regression model for HEYL mRNA levels and other clinical indexes in our internal cohort. The variable Hazard Ratio with 95%CI generated by Cox test were presented by liner point plot. (D) HEYL mRNA levels were quantified in 117 pairs of gastric cancer tissues and adjacent nontumor gastric tissues. (E) Kaplan‐Meier curves of overall survival in 117 gastric cancer patients validated by HEYL mRNA expression levels. (F) The comparison of diagnostic efficacy of CA19‐19 and HEYL mRNA level for tumor OS, stage and recurrence. Log‐rank tests were used to determine statistical significance in (E). (D) The results are shown as the mean ± SEM. *P < .05; **P < .01; ***P < .001

The preceding results mentioned the biological functions of risk TFs in TME component rebuilding and cancer‐related pathways activating. However, it is worthwhile to point out the key regulator especially participate in gastric cancer carcinogenesis. As a result, ten of the high‐risk TFs (Figure 1B, red highlighted) were shown significantly correlation with tumor stage indicated their underlying biological function both on tumor and TME cells. Meanwhile, the Gene Set Cancer Analysis (GSCALite) webserver was used to characterize the cancerous pathway activity of selected TFs and hes related family bHLH transcription factor with YRPW motif like (HEYL) performed outstanding percentage in association with EMT (Figure S1). Furthermore, in the TCGA‐STAD cohort, the samples with upregulated HEYL represented high enrichment of EMT, TGF‐β and Wnt/beta‐catenin pathways (GSEA analysis, *P* < .05; Figure 4A).

To explore whether the expression pattern of HEYL was an independent prognostic factor in gastric cancer, we carried out multivariate Cox analyses on HEYL expression values and other clinical information (age, gender, tumor stage, number of lymph node metastasis, mutation burden, and tumor subtype) as covariates in TCGA‐STAD cohort. Our results showed that the expression levels of HEYL and tumor stage III, IV were independent prognostic risk factors which correlated with patients’ dismal survival (*P* < .05, Figure 4B). The GC individual with a high expression level of HEYL in tumors harbored a 1.94‐fold high risk of death (*P* < .001; 95% CI, 1.302‐2.90). Multivariate results implied that the expression level of HEYL, tumor stage, and tumor size represented the approximate power to estimating GC patients’ prognosis (*P* < .05, Figure 4C). Adequately, we further validated the expression levels of HEYL in our internal 117 GC paired tissues, the HEYL was significantly upregulated in tumor samples compared with normal gastric tissues at the mRNA levels (*P* < .001; Figure 4D). The survival analysis demonstrated that GC patients with high expression level of HEYL indicated dismal survival outcomes for OS and DFS (*P* < .05; Figure 4E, Figure S2).

We then explored the significance of HEYL on clinicopathological prediction comparing with another GC serum biomarker CA19‐9. In bioproven GC individuals, the HEYL mRNA and serum CA19‐9 levels (kU/L) were used to construct a ROC curve to fully calculate the predicting accuracy for GC stage, OS, and tumor recurrence estimation. As a result, HEYL exhibits higher diagnostic power than CA19‐9 for patient's recurrence risk prediction (*P* < .05; Figure 4F). In conclusion, the HEYL is a potential prognostic and diagnostic biomarker for gastric cancer, and high expression of HEYL correlates with the activation of oncogenic signaling pathways in tumor.

### 3.5 HEYL exerts oncogenic activities in gastric cancer in vitro and in vivo

**Figure 5:**
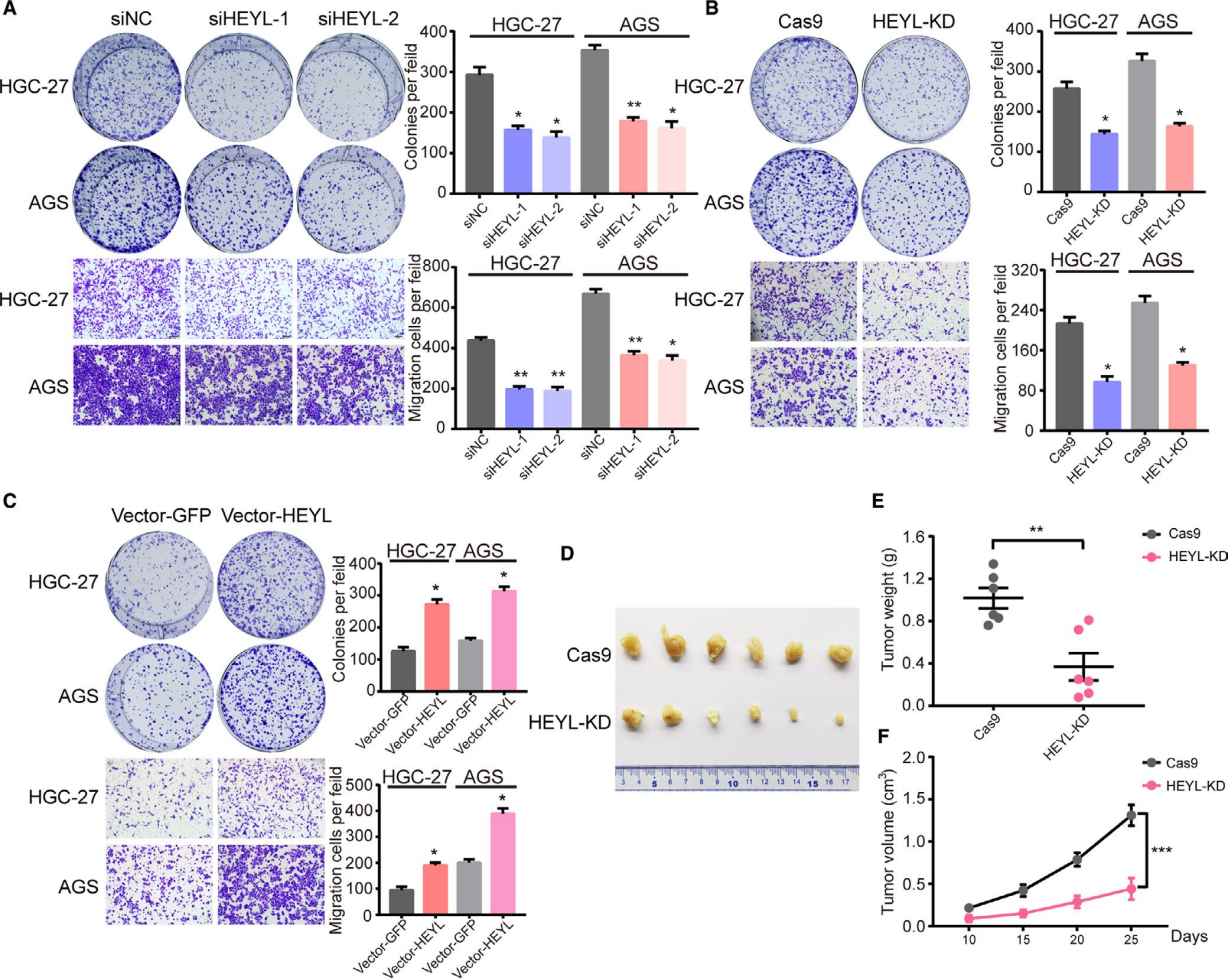
HEYL exerts oncogenic activities in GC. (A) Colony formation assays (up) and transwell migration assays (down) for HGC‐27 and AGS cells transfected with HEYL siRNAs or negative control siRNA. (B) Colony formation assays (up) and transwell migration assays (down) for HGC‐27 and AGS cells infected with the HEYL knockdown sgRNAs or control sgRNA lentivirus. (C) Colony formation assays (up) and transwell migration assays (down) for HGC‐27 and AGS cells infected with HEYL overexpression lentivirus or GFP control. (D) Xenograft tumors in nude mice. (E‐F) The knockdown of HEYL reduced the (E) weight and (F) volume of xenograft tumors (n = 6 mice per group). Values represent the mean ± SEM, (A‐C) n = 3 and (D‐F) n = 6. *P < .05; **P < .01; ***P < .001

Our former investigations have revealed the correlation between HEYL expression with TME components and clinical significations in gastric cancer, whereas absently known about the detail molecular function of HEYL in gastric cancer cell behaviors. We first utilized two independent siRNAs targeting HEYL (Figure S3A, Table S3) and found knockdown of HEYL markedly restrained proliferation and migration of these gastric cancer cells (Figure 5A). Consistently, the stably knockdown of HEYL in cells HGC‐27 and AGS dramatically inhibited proliferation and migration property using clustered regularly interspaced short palindromic repeats (CRISPR) deletion systems (Figure S3B, Figure 5B, Table S3). Overexpression of HEYL significantly promotes the carcinogenic process in vitro (Figure 5C; Figure S3C). To detect the significance of HEYL on tumorigenicity, HEYL deficient and control AGS cells were subcutaneously injected into the flanks of 6‐week‐old nude mice, which were monitored closely for tumor growth for nearly 4 weeks. Notably, knockdown of HEYL level observably decrease tumorigenicity in nude mice (Figure 5D), as determined by tumor weight (Figure 5E) and tumor size (Figure 5F). Collectively, these results manifest that HEYL promotes gastric cancer cell metastasis in vitro and growth both in vitro and in vivo.

### 3.6 HEYL regulates CDH11 expression under transcriptional level

**Figure 6:**
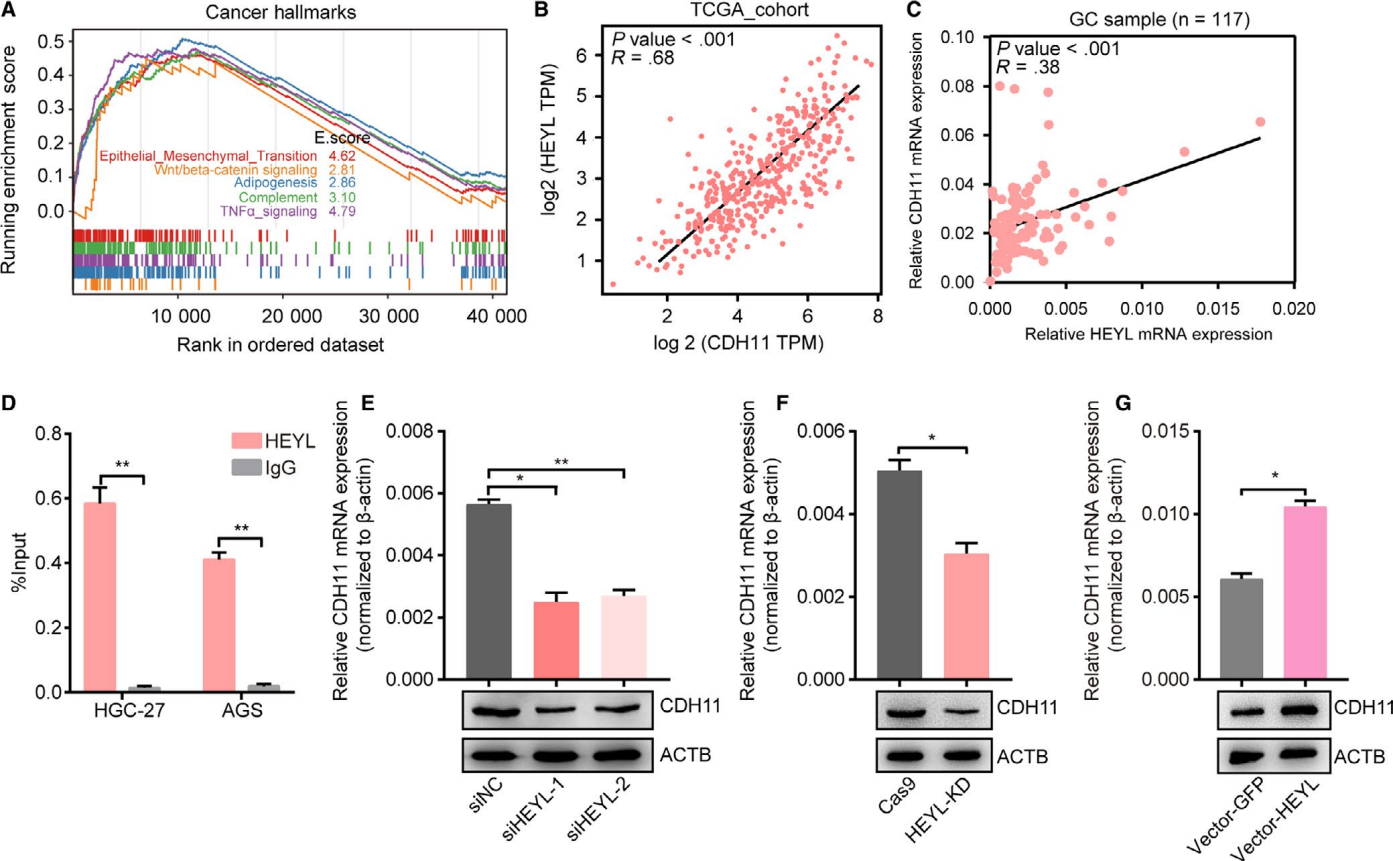
HEYL regulates CDH11 expression under the transcriptional level. (A) Cancer hallmark enrichment of downexpressed genes for AGS cells infected with HEYL knockdown sgRNAs or negative control. (B‐C) Expressional correlation between HEYL and CDH11 under mRNA levels in TCGA‐STAD database and GC samples. (D) ChIP‐qPCR validation for HEYL enrichment on CDH11 promoter in HGC‐27 and AGS cells. (E) RT‐qPCR and immunoblotting for CDH11 mRNA expression levels and protein levels transfected with HEYL siRNAs or control siRNA in AGS cells. (F) RT‐qPCR and immunoblotting for CDH11 mRNA expression levels and protein levels infected with HEYL knockdown sgRNAs or control sgRNA lentivirus in AGS cells. (G) RT‐qPCR and immunoblotting for CDH11 mRNA expression levels and protein levels infected with HEYL overexpression lentivirus or GFP control. (D‐G) Values represent the mean ± SEM, n = 3. *P < .05; **P < .01

To evaluate the contributions of HEYL for gastric cancer progression, we conducted HEYL knockdown AGS cells to undergo RNA sequencing (RNA‐seq) technology. We identified 229 upregulated genes and 297 downregulated genes (fold‐change >2). In order to explore the connection of HEYL expression with signaling pathways, we arranged the RNA‐seq profile and found that knockdown of HEYL is positively correlated with EMT, Wnt/beta‐catenin pathways (Figure 6A). Meanwhile, we analyzed the expressional correlation inside RNA‐seq dataset to select potential downstream targets of HEYL. The downregulated targets influenced by HEYL were chosen and undertake to the analysis of expressional correlation under their mRNA levels with HEYL mRNA levels in TCGA‐STAD database (Figure S4). Among them, CDH11 exhibits one of the highest expressional correlation with HEYL (R = 0.68; Figure 6B), and we also found their positively expressional correlation in our GC cohort (R = 0.38; Figure 6C). To authenticate the expressional regulation under transcriptional level, we designed the specific primers targeting CDH11 promoter. As a result, we found HEYL was enriched on the CDH11 promoter (Figure 6D). Using real‐time PCR and immunoblotting analyses, we also uncovered knockdown of HEYL decreased the mRNA and protein levels of CDH11 in AGS cells (Figure 6E‐F), and overexpression of HEYL upregulates CDH11 mRNA levels and protein levels (Figure 6G, Table S4). Taken together, these data suggest CDH11 as a downstream target of HEYL in gastric cancers.

### 3.7 CDH11 correlates with poor outcomes and promotes gastric cancer progression

**Figure 7:**
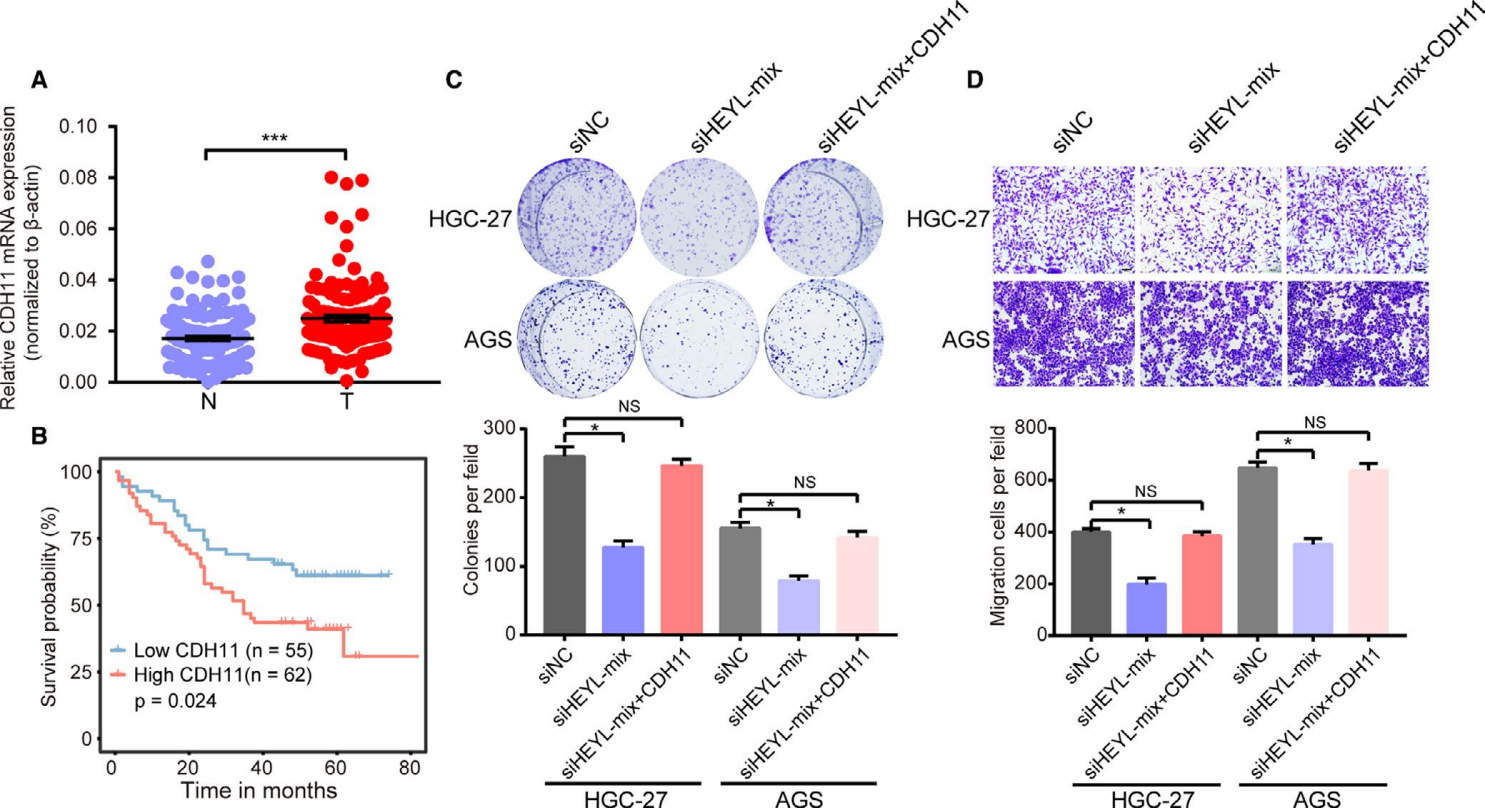
CDH11 correlates with poor outcomes and promotes gastric cancer progression in vitro. (A) CDH11 mRNA levels were quantified in 117 pairs of gastric tissues and adjacent nontumor tissues. (B) Kaplan‐Meier curves of overall survival in 117 gastric patients validated by CDH11 mRNA expression levels. (C‐D) (C) Colony formation assays and (D) Transwell migration assays for HGC‐27 and AGS cells transfected with HEYL siRNA mix, HEYL siRNA mix plus CDH11 overexpression plasmids or negative control siRNA. (B) Log‐rank tests were used to determine statistical significance. (A; C‐D) The results are shown as the mean ± SEM. *P < .05; ***P < .001

Cadherin 11 (CDH11) encodes a transmembrane protein, regulates cell fate decisions ^13^ and extracellular matrix synthesis.^14^ In human cancer, CDH11 promotes tumor development via controlling NF‐κB, EMT, and Wnt/beta‐catenin pathways.^15^, ^16^, ^17^ Several studies have highlighted the clinical implications and molecular functions of CDH11 in gastric cancer. We also searched the mRNA expression of CDH11 in our independent cohort of gastric tissues and found CDH11 was upregulated in gastric samples compared with adjacent nontumor gastric tissues (Figure 7A). Additionally, the upregulation of CDH11 positively correlated with the patient's poor OS and DFS rate (*P* < .05; Figure 7B, Figure S5). Importantly, overexpression of CDH11 could rescue the malignant behavior of HEYL‐knockdown gastric cancer cells (Figure 7C‐D). Collectively, these data demonstrate that CDH11 was upregulated in gastric cancer and as an oncogene to promote gastric cancer progression via transcriptional regulation by HEYL.

### 3.8 Discussions

The tumor stromal constituents, including stroma‐related cells, proteins, and signaling pathways, represented as vital fractions for tumor initiation, progression, and metastasis.^11^, ^18^, ^19^ Cancer development and progression onset in concert with transformation in the circumambient stroma, and tumor cells can functionally change their microenvironment through the secretion of various cytokines, chemokines, and other factors.^20^, ^21^, ^22^ The interaction between cancer cells and the surrounding stromal components eventually results in an environment that promotes tumor development and progression.

Several studies have shown that transcription factors were aberrantly overexpressed in diverse human cancers which act as oncogenes for tumor development and TME remodeling, and have recently emerged as attractive targets for cancer therapy.^23^, ^24^ For instance, STAT3, Snail, ZEB1, and Twist1 were described as epithelial‐to‐mesenchymal transition transcription factors (EMT‐TFs). These TFs involved in controlling global plasticity programs, affecting cell stemness and cell fate, and promoting coordinated cell plasticity changes in tumor parenchyma and stroma, ultimately, accelerated tumor metastasis and led to poor cancer prognosis.^25^, ^26^, ^27^ The new information suggested that the underlying regulatory TFs can be envisioned as essential metastasis and carcinogenesis‐promoting molecules, thereby enabling coordinated plasticity programs in parenchyma and stroma compartments in TME. We carried out a combination of transcriptomic analysis for TME subtypes estimation on TCGA‐STAD cohort and revealed a functional TME regulator HEYL which correlated with stromal component accumulation and activation of cancer‐related signatures in gastric cancer.

HEYL was identified as a basic helix‐loop‐helix transcription factor and a direct target of Notch signaling that promotes neuronal differentiation in neural stem cells and specifically correlates with EMT in heart tissue.^28^, ^29^ HEYL could also form heterodimeric complexes with HES1, an oncogene in many cancer types including gastric cancer, to regulate gene expression under transcription level.^30^ We first identified HEYL was upregulated in gastric cancer tissues and correlated with poor outcomes for GC patients. We also uncovered HEYL represents a promising diagnostic biomarker, compared with traditional biomarker CA19‐9 in gastric cancer recurrence. Meanwhile, molecular functions revealed HEYL promotes GC carcinogenesis via regulating CDH11 under the DNA level.

Previous studies have demonstrated CDH11 controls cell fated decisions and promotes cancer development. In our works, CDH11 is also highly expressed in GC tissues and represents poor survival of GC patients. Furthermore, CDH11 was regulated by HEYL and rescued oncogenic behavior in HEYL deficient gastric cancer cells. Importantly, emerging evidence has found CDH11 was significantly increased in the human tumoral stroma and regulates collagen and elastin synthesis.^14^, ^31^ Therefore, elucidating the crucial functions and regulatory mechanism of CDH11 in tumor stromal cells in future studies is important for understanding the progression of gastric cancer.

## 4 CONCLUSIONS

In this study, we first represent the quantified TME infiltration pattern remodeling by TFs in TCGA‐STAD cohort. Notably, we found HEYL is upregulated and correlated with dismal prognosis in gastric cancer patients. Meanwhile, HEYL enhances carcinogenesis and metastasis through activating cancer‐related signaling pathways by regulating CDH11 expression level in GC cells. Our findings demonstrated the important roles of HEYL in GC progression and indicated HEYL represents a potential prognostic and therapeutic target in gastric cancer.

## CONFLICT OF INTEREST

No potential conflicts of interest are disclosed.

## AUTHOR CONTRIBUTIONS

WW and ZQ designed the study; HL and SN acquired the data; NS and HL performed the analysis of data. HL and HW wrote the paper with comments from all authors.

## ETHICAL STATEMENT

This study was approved by the Ethics Committee of Shanghai Medical College of Fudan University.

## Supporting information

Table S1‐S4

Supplementary Material

## Data Availability

The datasets performed in this study are available from the corresponding author on reasonable request.

## References

[1] Poorolajal J , Moradi L , Mohammadi Y , Cheraghi Z , Gohari‐Ensaf F . Cheraghi Z, Gohari‐Ensaf F. Risk factors for stomach cancer: a systematic review and meta‐analysis. Epidemiol. Health. 2020:42;e2020004. 10.4178/epih.e2020004 PMC7056944 32023777

[2] den Hoed CM , Kuipers EJ . Gastric Cancer: How Can We Reduce the Incidence of this Disease? Curr Gastroenterol Rep. 2016;18:34. 27184043 10.1007/s11894-016-0506-0 PMC4868864

[3] Zhou ZH , Ji CD , Xiao HL , Zhao HB , Cui YH , Bian XW . Reorganized Collagen in the Tumor Microenvironment of Gastric Cancer and Its Association with Prognosis. J Cancer. 2017;8:1466‐1476. 28638462 10.7150/jca.18466 PMC5479253

[4] Chen Y , Zhang S , Wang Q , Zhang X . Tumor‐recruited M2 macrophages promote gastric and breast cancer metastasis via M2 macrophage‐secreted CHI3L1 protein. J Hematol Oncol. 2017;10:36. 28143526 10.1186/s13045-017-0408-0 PMC5286803

[5] Kim J , Bae JS . Tumor‐Associated Macrophages and Neutrophils in Tumor Microenvironment. Mediators Inflamm. 2016;2016:6058147. 26966341 10.1155/2016/6058147 PMC4757693

[6] Son B , Lee S , Youn H , Kim E , Kim W , Youn B . The role of tumor microenvironment in therapeutic resistance. Oncotarget. 2017;8:3933‐3945. 27965469 10.18632/oncotarget.13907 PMC5354804

[7] Ma B , Wells A , Clark AM . The pan‐therapeutic resistance of disseminated tumor cells: Role of phenotypic plasticity and the metastatic microenvironment. Semin Cancer Biol. 2019. 10.1016/j.semcancer.2019.07.021 PMC6992520 31376430

[8] Li H , Fan X , Houghton J . Tumor microenvironment: the role of the tumor stroma in cancer. J Cell Biochem. 2007;101:805‐815. 17226777 10.1002/jcb.21159

[9] Johnston SJ , Carroll JS . Transcription factors and chromatin proteins as therapeutic targets in cancer. Biochim Biophys Acta. 2015;1855:183‐192. 25721328 10.1016/j.bbcan.2015.02.002

[10] Tania M , Khan MA , Fu J . Epithelial to mesenchymal transition inducing transcription factors and metastatic cancer. Tumour Biol. 2014;35:7335‐7342. 24880591 10.1007/s13277-014-2163-y

[11] Baulida J . Epithelial‐to‐mesenchymal transition transcription factors in cancer‐associated fibroblasts. Mol Oncol. 2017;11:847‐859. 28544627 10.1002/1878-0261.12080 PMC5496490

[12] McCarthy JB , El‐Ashry D , Turley EA . Hyaluronan, cancer‐associated fibroblasts and the tumor microenvironment in malignant progression. Front Cell Dev Biol. 2018;6:48. 29868579 10.3389/fcell.2018.00048 PMC5951929

[13] Alimperti S , Andreadis ST . CDH2 and CDH11 act as regulators of stem cell fate decisions. Stem Cell Res. 2015;14:270‐282. 25771201 10.1016/j.scr.2015.02.002 PMC4439315

[14] Row S , Liu Y , Alimperti S , Agarwal SK , Andreadis ST . Cadherin‐11 is a novel regulator of extracellular matrix synthesis and tissue mechanics. J Cell Sci. 2016;129:2950‐2961. 27311482 10.1242/jcs.183772 PMC5004872

[15] Satriyo PB , Bamodu OA , Chen JH , et al. Cadherin 11 Inhibition Downregulates beta‐catenin, Deactivates the Canonical WNT Signalling Pathway and Suppresses the Cancer Stem Cell‐Like Phenotype of Triple Negative Breast Cancer. J Clin Med. 2019;8(2):148–164. 10.3390/jcm8020148 PMC6407101 30691241

[16] Yao J , Deng B , Zheng L , Dou L , Guo Y , Guo K . miR‐27b is upregulated in cervical carcinogenesis and promotes cell growth and invasion by regulating CDH11 and epithelial‐mesenchymal transition. Oncol Rep. 2016;35:1645‐1651. 26706910 10.3892/or.2015.4500

[17] Zhang JX , He WL , Feng ZH , et al. A positive feedback loop consisting of C12orf59/NF‐kappaB/CDH11 promotes gastric cancer invasion and metastasis. J Exp Clin Cancer Res. 2019;38:164. 30987656 10.1186/s13046-019-1114-2 PMC6463669

[18] Wang M , Zhang J , Huang Y , et al. Cancer‐associated fibroblasts autophagy enhances progression of triple‐negative breast cancer cells. Med Sci Monit. 2017;23:3904‐3912. 28802099 10.12659/MSM.902870 PMC5565237

[19] Wu X , Tao P , Zhou Q , et al. IL‐6 secreted by cancer‐associated fibroblasts promotes epithelial‐mesenchymal transition and metastasis of gastric cancer via JAK2/STAT3 signaling pathway. Oncotarget. 2017;8:20741‐20750. 28186964 10.18632/oncotarget.15119 PMC5400541

[20] Michea P , Noël F , Zakine E , et al. Adjustment of dendritic cells to the breast‐cancer microenvironment is subset specific. Nat Immunol. 2018;19:885‐897. 30013147 10.1038/s41590-018-0145-8

[21] Yang L , Zhang Y . Tumor‐associated macrophages: from basic research to clinical application. J Hematol Oncol. 2017;10:58. 28241846 10.1186/s13045-017-0430-2 PMC5329931

[22] Hinshaw DC , Shevde LA . The tumor microenvironment innately modulates cancer progression. Cancer Res. 2019;79:4557‐4566. 31350295 10.1158/0008-5472.CAN-18-3962 PMC6744958

[23] Wu ZZ , Chen LS , Zhou R , Bin JP , Liao YL , Liao WJ . Metastasis‐associated in colon cancer‐1 in gastric cancer: Beyond metastasis. World J Gastroenterol. 2016;22:6629‐6637. 27547006 10.3748/wjg.v22.i29.6629 PMC4970472

[24] Pan Z , Tian Y , Zhang B , et al. YAP signaling in gastric cancer‐derived mesenchymal stem cells is critical for its promoting role in cancer progression. Int J Oncol. 2017;51:1055‐1066. 28848999 10.3892/ijo.2017.4101 PMC5592864

[25] Lee KW , Yeo SY , Sung CO , Kim SH . Twist1 is a key regulator of cancer‐associated fibroblasts. Cancer Res. 2015;75:73‐85. 25368021 10.1158/0008-5472.CAN-14-0350

[26] Skrypek N , Goossens S , De Smedt E , Vandamme N , Berx G . Epithelial‐to‐Mesenchymal Transition: Epigenetic Reprogramming Driving Cellular Plasticity. Trends Genet. 2017;33:943‐959. 28919019 10.1016/j.tig.2017.08.004

[27] Ye X , Tam WL , Shibue T , et al. Distinct EMT programs control normal mammary stem cells and tumour‐initiating cells. Nature. 2015;525:256‐260. 26331542 10.1038/nature14897 PMC4764075

[28] Fischer A , Steidl C , Wagner TU , et al. Combined loss of Hey1 and HeyL causes congenital heart defects because of impaired epithelial to mesenchymal transition. Circ Res. 2007;100:856‐863. 17303760 10.1161/01.RES.0000260913.95642.3b

[29] Hirano K , Namihira M . LSD1 Mediates Neuronal Differentiation of Human Fetal Neural Stem Cells by Controlling the Expression of a Novel Target Gene. HEYL. Stem Cells. 2016;34:1872‐1882. 27018646 10.1002/stem.2362

[30] Noguchi Y‐T , Nakamura M , Hino N , et al. Cell‐autonomous and redundant roles of Hey1 and HeyL in muscle stem cells: HeyL requires Hes1 to bind diverse DNA sites. Development. 2019;146(4).dev163618. 10.1242/dev.163618 30745427

[31] Torres S , Bartolome RA , Mendes M , et al. Proteome profiling of cancer‐associated fibroblasts identifies novel proinflammatory signatures and prognostic markers for colorectal cancer. Clin Cancer Res. 2013;19:6006‐6019. 24025712 10.1158/1078-0432.CCR-13-1130

