# Supplementary material for: Charactering tumor microenvironment reveals stromal‐related transcription factors promote tumor carcinogenesis in gastric cancer
Source: Cancer Med. 2020 May 28;9(14):5247–57. doi: 10.1002/cam4.3133 (PMC7367614; doi:10.1002/cam4.3133)
Supplement: Supplementary file 1 — Table S1‐S4 [file CAM4-9-5247-s001.docx]

**Supporting table S1. The up and down-regulated TFs in TCGA-STAD cohort.**

| **Gene.id** | **LogFC** | **AveExpr** | **Adj.P.Val** | **Log.rank.p** | **Uni.cox.p** | **HR** | **HR.upper** | **HR.low** |
| --- | --- | --- | --- | --- | --- | --- | --- | --- |
| MAZ | 8.042E-01 | 4.708E+00 | 8.427E-06 | 1.584E-02 | 1.650E-02 | 6.710E-01 | 9.300E-01 | 4.840E-01 |
| ZNF680 | 3.386E-01 | 1.167E+00 | 3.430E-02 | 9.992E-02 | 1.010E-01 | 7.470E-01 | 1.060E+00 | 5.280E-01 |
| FOSL1 | 1.087E+00 | 3.114E+00 | 3.770E-02 | 2.839E-01 | 2.840E-01 | 8.270E-01 | 1.170E+00 | 5.850E-01 |
| BATF | 1.246E+00 | 2.656E+00 | 2.646E-03 | 8.299E-02 | 8.420E-02 | 1.340E+00 | 1.880E+00 | 9.610E-01 |
| ZNF789 | 4.805E-01 | 1.031E+00 | 4.470E-04 | 9.329E-02 | 9.440E-02 | 7.560E-01 | 1.050E+00 | 5.450E-01 |
| ZNF239 | 7.242E-01 | 1.285E+00 | 5.445E-05 | 1.992E-01 | 2.000E-01 | 7.930E-01 | 1.130E+00 | 5.570E-01 |
| YBX1 | 3.676E-01 | 7.972E+00 | 1.889E-02 | 5.471E-02 | 5.580E-02 | 7.220E-01 | 1.010E+00 | 5.170E-01 |
| MBD3 | 3.777E-01 | 2.720E+00 | 1.098E-02 | 5.770E-02 | 5.880E-02 | 7.170E-01 | 1.010E+00 | 5.080E-01 |
| MSX1 | 6.203E-01 | 1.459E+00 | 1.409E-02 | 4.365E-02 | 4.500E-02 | 6.750E-01 | 9.910E-01 | 4.600E-01 |
| ZNF205 | 6.036E-01 | 2.613E+00 | 5.520E-05 | 5.108E-02 | 5.210E-02 | 7.220E-01 | 1.000E+00 | 5.200E-01 |
| ZNF592 | 5.011E-01 | 3.059E+00 | 1.777E-03 | 3.494E-01 | 3.500E-01 | 1.170E+00 | 1.630E+00 | 8.410E-01 |
| ZNF785 | 3.357E-01 | 1.391E+00 | 9.767E-03 | 6.175E-01 | 6.180E-01 | 1.090E+00 | 1.540E+00 | 7.750E-01 |
| CTCF | 4.519E-01 | 3.794E+00 | 1.802E-05 | 4.124E-01 | 4.130E-01 | 1.150E+00 | 1.630E+00 | 8.180E-01 |
| ZNF692 | 5.340E-01 | 2.000E+00 | 2.915E-03 | 9.580E-02 | 9.740E-02 | 7.250E-01 | 1.060E+00 | 4.950E-01 |
| TGIF1 | 9.140E-01 | 2.699E+00 | 1.939E-07 | 1.274E-01 | 1.280E-01 | 7.750E-01 | 1.080E+00 | 5.590E-01 |
| IRF9 | 3.911E-01 | 1.239E+00 | 4.760E-02 | 1.953E-01 | 1.970E-01 | 7.930E-01 | 1.130E+00 | 5.580E-01 |
| CDC5L | 3.769E-01 | 2.980E+00 | 1.765E-03 | 3.688E-02 | 3.850E-02 | 1.550E+00 | 2.340E+00 | 1.020E+00 |
| ZNF689 | 3.539E-01 | 2.067E+00 | 4.176E-02 | 7.274E-02 | 7.420E-02 | 7.090E-01 | 1.030E+00 | 4.870E-01 |
| ZNF195 | 7.605E-01 | 1.837E+00 | 3.063E-07 | 1.005E-01 | 1.020E-01 | 7.620E-01 | 1.060E+00 | 5.500E-01 |
| SART1 | 4.894E-01 | 3.927E+00 | 5.069E-04 | 1.366E-01 | 1.380E-01 | 7.660E-01 | 1.090E+00 | 5.390E-01 |
| ZBTB12 | 6.422E-01 | 1.675E+00 | 2.360E-03 | 2.319E-01 | 2.330E-01 | 8.090E-01 | 1.150E+00 | 5.710E-01 |
| ZNF687 | 5.580E-01 | 3.082E+00 | 1.406E-04 | 1.739E-01 | 1.750E-01 | 7.940E-01 | 1.110E+00 | 5.690E-01 |
| ZNF263 | 3.753E-01 | 1.997E+00 | 2.880E-03 | 8.005E-02 | 8.150E-02 | 7.190E-01 | 1.040E+00 | 4.960E-01 |
| LCOR | 7.170E-01 | 2.450E+00 | 2.314E-03 | 1.754E-02 | 1.860E-02 | 6.240E-01 | 9.240E-01 | 4.210E-01 |
| ZNF841 | 4.563E-01 | 1.578E+00 | 9.617E-03 | 3.018E-01 | 3.020E-01 | 8.170E-01 | 1.200E+00 | 5.560E-01 |
| ZNF816 | 4.825E-01 | 1.472E+00 | 2.257E-03 | 1.871E-01 | 1.880E-01 | 7.740E-01 | 1.130E+00 | 5.290E-01 |
| TFDP1 | 8.121E-01 | 4.510E+00 | 4.216E-07 | 9.354E-04 | 1.090E-03 | 5.630E-01 | 7.950E-01 | 3.990E-01 |
| MNX1 | 9.958E-01 | 1.110E+00 | 3.278E-05 | 3.026E-03 | 3.340E-03 | 6.120E-01 | 8.490E-01 | 4.400E-01 |
| HNF4A | 2.048E+00 | 3.294E+00 | 1.122E-03 | 8.313E-03 | 8.850E-03 | 6.430E-01 | 8.950E-01 | 4.620E-01 |
| FOXS1 | 1.402E+00 | 1.281E+00 | 1.698E-08 | 3.599E-03 | 4.000E-03 | 1.690E+00 | 2.430E+00 | 1.180E+00 |
| ZBTB39 | 4.318E-01 | 1.506E+00 | 4.060E-03 | 9.115E-02 | 9.210E-02 | 7.390E-01 | 1.050E+00 | 5.190E-01 |
| PATZ1 | 6.535E-01 | 2.835E+00 | 1.363E-03 | 2.312E-01 | 2.320E-01 | 7.940E-01 | 1.160E+00 | 5.440E-01 |
| STAT2 | 6.704E-01 | 3.426E+00 | 2.757E-03 | 3.209E-01 | 3.210E-01 | 8.420E-01 | 1.180E+00 | 6.000E-01 |
| ZNF668 | 4.292E-01 | 1.237E+00 | 2.388E-04 | 2.001E-01 | 2.010E-01 | 7.960E-01 | 1.130E+00 | 5.620E-01 |
| ZNF611 | 3.830E-01 | 1.189E+00 | 1.332E-03 | 9.185E-02 | 9.280E-02 | 7.440E-01 | 1.050E+00 | 5.270E-01 |
| FBXO41 | 8.449E-01 | 1.195E+00 | 6.326E-06 | 1.558E-01 | 1.570E-01 | 7.570E-01 | 1.110E+00 | 5.150E-01 |
| FOXM1 | 2.257E+00 | 2.924E+00 | 9.756E-10 | 2.309E-02 | 2.400E-02 | 6.650E-01 | 9.480E-01 | 4.660E-01 |
| ZNF468 | 8.884E-01 | 1.932E+00 | 5.632E-06 | 1.486E-01 | 1.500E-01 | 7.490E-01 | 1.110E+00 | 5.060E-01 |
| MBD4 | 3.284E-01 | 3.074E+00 | 4.451E-02 | 1.247E-01 | 1.250E-01 | 7.750E-01 | 1.070E+00 | 5.590E-01 |
| NFYA | 6.265E-01 | 2.867E+00 | 1.337E-05 | 3.804E-01 | 3.800E-01 | 8.580E-01 | 1.210E+00 | 6.080E-01 |
| ZNF35 | 3.370E-01 | 1.874E+00 | 1.194E-03 | 1.604E-01 | 1.610E-01 | 7.830E-01 | 1.100E+00 | 5.570E-01 |
| ZNF320 | 4.532E-01 | 1.500E+00 | 4.684E-03 | 1.847E-01 | 1.860E-01 | 7.670E-01 | 1.140E+00 | 5.180E-01 |
| ZNF296 | 8.085E-01 | 2.419E+00 | 1.933E-02 | 3.069E-02 | 3.160E-02 | 6.870E-01 | 9.680E-01 | 4.880E-01 |
| ZNF526 | 5.467E-01 | 2.172E+00 | 5.253E-05 | 2.567E-01 | 2.570E-01 | 8.140E-01 | 1.160E+00 | 5.710E-01 |
| TBX2 | 4.717E-01 | 1.974E+00 | 2.691E-02 | 1.639E-01 | 1.650E-01 | 1.260E+00 | 1.750E+00 | 9.090E-01 |
| ZNF28 | 6.170E-01 | 1.598E+00 | 7.840E-04 | 9.498E-02 | 9.600E-02 | 7.560E-01 | 1.050E+00 | 5.440E-01 |
| GMEB2 | 5.844E-01 | 2.581E+00 | 7.933E-06 | 7.536E-02 | 7.660E-02 | 7.320E-01 | 1.030E+00 | 5.180E-01 |
| ZNF182 | 4.087E-01 | 1.306E+00 | 4.526E-03 | 1.240E-01 | 1.250E-01 | 1.350E+00 | 1.970E+00 | 9.200E-01 |
| ZNF101 | 4.151E-01 | 1.509E+00 | 1.055E-02 | 1.921E-04 | 2.400E-04 | 5.400E-01 | 7.500E-01 | 3.880E-01 |
| KLF16 | 1.166E+00 | 3.838E+00 | 3.336E-06 | 2.402E-02 | 2.520E-02 | 6.600E-01 | 9.490E-01 | 4.580E-01 |
| MYBL2 | 2.893E+00 | 3.751E+00 | 1.622E-09 | 2.913E-01 | 2.920E-01 | 8.390E-01 | 1.160E+00 | 6.060E-01 |
| EP300 | 3.555E-01 | 4.006E+00 | 3.062E-02 | 9.183E-02 | 9.270E-02 | 7.560E-01 | 1.050E+00 | 5.450E-01 |
| HOXB9 | 1.689E+00 | 1.400E+00 | 3.653E-06 | 1.262E-01 | 1.270E-01 | 7.570E-01 | 1.080E+00 | 5.290E-01 |
| ZNF664 | 4.279E-01 | 4.170E+00 | 2.361E-02 | 6.088E-02 | 6.190E-02 | 7.340E-01 | 1.020E+00 | 5.300E-01 |
| FOXJ1 | 1.575E+00 | 1.202E+00 | 1.441E-05 | 8.561E-02 | 8.680E-02 | 7.420E-01 | 1.040E+00 | 5.270E-01 |
| HOXC10 | 2.375E+00 | 1.274E+00 | 1.924E-08 | 6.054E-02 | 6.170E-02 | 7.190E-01 | 1.020E+00 | 5.090E-01 |
| FIZ1 | 4.252E-01 | 1.923E+00 | 5.348E-04 | 3.988E-02 | 4.110E-02 | 6.880E-01 | 9.850E-01 | 4.800E-01 |
| SMARCC1 | 6.627E-01 | 3.721E+00 | 1.043E-05 | 7.585E-02 | 7.750E-02 | 7.020E-01 | 1.040E+00 | 4.740E-01 |
| MYRF | 1.303E+00 | 3.098E+00 | 1.745E-02 | 1.228E-01 | 1.240E-01 | 7.530E-01 | 1.080E+00 | 5.250E-01 |
| SP100 | 3.502E-01 | 2.645E+00 | 4.303E-02 | 2.719E-02 | 2.810E-02 | 1.440E+00 | 1.990E+00 | 1.040E+00 |
| ZNF384 | 3.389E-01 | 2.834E+00 | 1.360E-02 | 9.732E-02 | 9.850E-02 | 7.530E-01 | 1.050E+00 | 5.380E-01 |
| FOXC1 | 1.410E+00 | 2.531E+00 | 7.588E-05 | 8.442E-02 | 8.580E-02 | 7.460E-01 | 1.040E+00 | 5.350E-01 |
| ZNF669 | 4.339E-01 | 1.528E+00 | 4.350E-03 | 3.967E-02 | 4.080E-02 | 6.890E-01 | 9.850E-01 | 4.820E-01 |
| ZBTB33 | 8.712E-01 | 2.665E+00 | 2.733E-05 | 2.003E-01 | 2.010E-01 | 7.840E-01 | 1.140E+00 | 5.400E-01 |
| GTF3A | 6.836E-01 | 4.729E+00 | 5.654E-04 | 2.734E-01 | 2.740E-01 | 8.340E-01 | 1.150E+00 | 6.030E-01 |
| ZNF202 | 3.889E-01 | 1.491E+00 | 1.972E-03 | 3.886E-02 | 4.040E-02 | 6.500E-01 | 9.810E-01 | 4.300E-01 |
| HIVEP3 | 3.532E-01 | 1.009E+00 | 1.785E-02 | 1.822E-01 | 1.830E-01 | 8.010E-01 | 1.110E+00 | 5.780E-01 |
| ZNF317 | 3.681E-01 | 2.849E+00 | 8.049E-04 | 7.311E-03 | 7.800E-03 | 6.390E-01 | 8.890E-01 | 4.590E-01 |
| ZNF251 | 4.394E-01 | 1.666E+00 | 2.494E-02 | 3.510E-01 | 3.520E-01 | 8.300E-01 | 1.230E+00 | 5.610E-01 |
| ZNF746 | 4.361E-01 | 1.906E+00 | 1.224E-04 | 2.790E-02 | 2.880E-02 | 6.830E-01 | 9.610E-01 | 4.860E-01 |
| ATF1 | 3.565E-01 | 3.142E+00 | 9.929E-03 | 7.515E-02 | 7.640E-02 | 7.130E-01 | 1.040E+00 | 4.910E-01 |
| GLI4 | 3.280E-01 | 2.147E+00 | 6.374E-03 | 9.804E-02 | 9.940E-02 | 7.520E-01 | 1.060E+00 | 5.360E-01 |
| NUFIP1 | 5.366E-01 | 1.628E+00 | 2.576E-06 | 5.491E-02 | 5.650E-02 | 6.620E-01 | 1.010E+00 | 4.330E-01 |
| CERS5 | 3.233E-01 | 2.054E+00 | 4.385E-03 | 7.024E-02 | 7.130E-02 | 1.350E+00 | 1.870E+00 | 9.740E-01 |
| ZNF473 | 5.760E-01 | 1.252E+00 | 7.658E-06 | 1.900E-01 | 1.910E-01 | 7.970E-01 | 1.120E+00 | 5.670E-01 |
| ZSCAN29 | 4.710E-01 | 1.958E+00 | 8.071E-04 | 2.932E-01 | 2.940E-01 | 8.210E-01 | 1.190E+00 | 5.680E-01 |
| HHEX | 8.886E-01 | 2.107E+00 | 1.313E-03 | 8.147E-02 | 8.300E-02 | 1.350E+00 | 1.900E+00 | 9.610E-01 |
| ZNF200 | 4.737E-01 | 1.015E+00 | 4.254E-08 | 1.607E-02 | 1.670E-02 | 6.720E-01 | 9.310E-01 | 4.860E-01 |
| TP53 | 7.465E-01 | 3.968E+00 | 3.327E-03 | 1.234E-02 | 1.310E-02 | 6.330E-01 | 9.080E-01 | 4.410E-01 |
| ETV7 | 1.028E+00 | 2.436E+00 | 7.438E-03 | 5.544E-02 | 5.650E-02 | 7.260E-01 | 1.010E+00 | 5.220E-01 |
| POU2F1 | 6.845E-01 | 1.819E+00 | 3.492E-06 | 2.445E-01 | 2.450E-01 | 8.180E-01 | 1.150E+00 | 5.840E-01 |
| ZNF629 | 5.563E-01 | 2.637E+00 | 5.316E-03 | 1.646E-01 | 1.650E-01 | 7.930E-01 | 1.100E+00 | 5.720E-01 |
| HOXA10 | 1.763E+00 | 1.162E+00 | 1.876E-09 | 5.110E-03 | 5.540E-03 | 6.160E-01 | 8.680E-01 | 4.380E-01 |
| ZNF232 | 4.032E-01 | 1.597E+00 | 6.946E-03 | 5.903E-02 | 5.990E-02 | 7.320E-01 | 1.010E+00 | 5.290E-01 |
| FOXN2 | 6.169E-01 | 2.289E+00 | 2.144E-04 | 2.032E-02 | 2.110E-02 | 6.810E-01 | 9.440E-01 | 4.910E-01 |
| ZNF777 | 4.491E-01 | 3.019E+00 | 4.726E-03 | 7.395E-02 | 7.520E-02 | 7.280E-01 | 1.030E+00 | 5.130E-01 |
| STAT3 | 4.202E-01 | 4.783E+00 | 1.235E-02 | 1.828E-02 | 1.910E-02 | 1.480E+00 | 2.040E+00 | 1.070E+00 |
| SP110 | 3.996E-01 | 1.148E+00 | 7.522E-03 | 4.032E-01 | 4.040E-01 | 1.150E+00 | 1.610E+00 | 8.260E-01 |
| MTA2 | 6.095E-01 | 4.535E+00 | 1.628E-04 | 2.522E-03 | 2.800E-03 | 6.060E-01 | 8.410E-01 | 4.360E-01 |
| ZSCAN16 | 5.155E-01 | 1.982E+00 | 8.626E-04 | 1.497E-02 | 1.560E-02 | 6.680E-01 | 9.260E-01 | 4.820E-01 |
| TULP4 | 5.195E-01 | 2.073E+00 | 1.318E-03 | 3.294E-01 | 3.310E-01 | 8.260E-01 | 1.210E+00 | 5.630E-01 |
| ZXDC | 3.629E-01 | 2.192E+00 | 1.856E-02 | 8.862E-02 | 8.970E-02 | 7.530E-01 | 1.040E+00 | 5.430E-01 |
| GMEB1 | 4.558E-01 | 1.797E+00 | 5.316E-06 | 3.577E-03 | 3.940E-03 | 5.990E-01 | 8.490E-01 | 4.230E-01 |
| RELB | 1.020E+00 | 3.323E+00 | 1.121E-04 | 8.256E-02 | 8.350E-02 | 7.460E-01 | 1.040E+00 | 5.360E-01 |
| ZFPM1 | 9.822E-01 | 3.291E+00 | 4.205E-03 | 2.517E-02 | 2.620E-02 | 6.740E-01 | 9.540E-01 | 4.760E-01 |
| NFKB1 | 5.057E-01 | 3.494E+00 | 5.358E-04 | 2.876E-02 | 2.980E-02 | 6.730E-01 | 9.620E-01 | 4.710E-01 |
| CEBPG | 5.446E-01 | 3.464E+00 | 7.370E-03 | 1.090E-01 | 1.100E-01 | 7.590E-01 | 1.060E+00 | 5.410E-01 |
| LZTS3 | 9.847E-01 | 2.663E+00 | 5.195E-03 | 7.004E-02 | 7.150E-02 | 7.010E-01 | 1.030E+00 | 4.760E-01 |
| ZNF768 | 4.975E-01 | 4.204E+00 | 3.332E-02 | 6.267E-02 | 6.370E-02 | 7.350E-01 | 1.020E+00 | 5.310E-01 |
| HEY1 | 5.026E-01 | 1.210E+00 | 2.456E-03 | 6.371E-02 | 6.500E-02 | 1.370E+00 | 1.930E+00 | 9.800E-01 |
| NFATC2 | 4.858E-01 | 2.528E+00 | 4.345E-02 | 1.752E-01 | 1.760E-01 | 7.980E-01 | 1.110E+00 | 5.760E-01 |
| ZNF551 | 3.526E-01 | 1.298E+00 | 4.050E-03 | 2.890E-01 | 2.900E-01 | 8.110E-01 | 1.200E+00 | 5.490E-01 |
| ELK1 | 8.416E-01 | 3.507E+00 | 3.391E-06 | 3.572E-01 | 3.580E-01 | 8.350E-01 | 1.230E+00 | 5.680E-01 |
| VPS72 | 5.283E-01 | 3.154E+00 | 1.758E-04 | 1.186E-01 | 1.200E-01 | 7.620E-01 | 1.070E+00 | 5.410E-01 |
| ETS1 | 1.045E+00 | 3.491E+00 | 5.938E-04 | 1.572E-01 | 1.580E-01 | 1.280E+00 | 1.820E+00 | 9.070E-01 |
| ZNF217 | 1.005E+00 | 3.035E+00 | 1.290E-04 | 3.717E-01 | 3.710E-01 | 8.540E-01 | 1.210E+00 | 6.050E-01 |
| IRF8 | 1.084E+00 | 2.627E+00 | 1.413E-02 | 5.567E-02 | 5.700E-02 | 6.910E-01 | 1.010E+00 | 4.720E-01 |
| BAZ2A | 5.107E-01 | 3.620E+00 | 8.727E-03 | 2.338E-01 | 2.350E-01 | 8.210E-01 | 1.140E+00 | 5.930E-01 |
| ZNF480 | 3.641E-01 | 2.012E+00 | 1.577E-02 | 5.388E-01 | 5.390E-01 | 1.130E+00 | 1.650E+00 | 7.690E-01 |
| ZNF574 | 4.447E-01 | 2.844E+00 | 2.975E-03 | 3.607E-01 | 3.610E-01 | 8.380E-01 | 1.220E+00 | 5.740E-01 |
| TOP3A | 5.480E-01 | 2.143E+00 | 7.516E-05 | 1.144E-01 | 1.160E-01 | 7.550E-01 | 1.070E+00 | 5.320E-01 |
| ZBTB45 | 3.763E-01 | 2.942E+00 | 5.246E-03 | 3.082E-01 | 3.090E-01 | 1.190E+00 | 1.650E+00 | 8.540E-01 |
| CARHSP1 | 5.763E-01 | 3.254E+00 | 1.407E-02 | 7.266E-02 | 7.390E-02 | 7.350E-01 | 1.030E+00 | 5.250E-01 |
| IRF7 | 1.162E+00 | 3.532E+00 | 1.083E-04 | 3.044E-01 | 3.050E-01 | 1.190E+00 | 1.650E+00 | 8.560E-01 |
| MTA1 | 3.897E-01 | 3.194E+00 | 1.080E-02 | 3.510E-02 | 3.610E-02 | 1.420E+00 | 1.970E+00 | 1.020E+00 |
| ZKSCAN5 | 3.243E-01 | 1.650E+00 | 1.619E-02 | 9.792E-02 | 9.950E-02 | 7.310E-01 | 1.060E+00 | 5.030E-01 |
| IRF5 | 5.919E-01 | 1.814E+00 | 3.752E-02 | 4.508E-01 | 4.520E-01 | 8.680E-01 | 1.260E+00 | 5.990E-01 |
| FOXK2 | 5.170E-01 | 2.773E+00 | 9.681E-05 | 2.902E-02 | 2.980E-02 | 6.970E-01 | 9.650E-01 | 5.030E-01 |
| TFAP4 | 8.083E-01 | 1.459E+00 | 1.304E-08 | 1.726E-02 | 1.800E-02 | 6.690E-01 | 9.340E-01 | 4.800E-01 |
| ERF | 7.243E-01 | 4.292E+00 | 2.961E-03 | 2.006E-01 | 2.020E-01 | 8.060E-01 | 1.120E+00 | 5.790E-01 |
| ZFP64 | 6.594E-01 | 2.162E+00 | 6.762E-06 | 3.287E-02 | 3.370E-02 | 6.950E-01 | 9.720E-01 | 4.970E-01 |
| CEBPB | 6.893E-01 | 6.011E+00 | 2.463E-02 | 6.468E-02 | 6.600E-02 | 1.440E+00 | 2.130E+00 | 9.760E-01 |
| STAT5A | 4.883E-01 | 2.743E+00 | 2.848E-02 | 8.059E-02 | 8.160E-02 | 7.400E-01 | 1.040E+00 | 5.270E-01 |
| RFX5 | 6.525E-01 | 3.430E+00 | 1.047E-03 | 1.764E-03 | 2.000E-03 | 5.810E-01 | 8.200E-01 | 4.110E-01 |
| ZNF22 | 4.195E-01 | 2.355E+00 | 4.411E-02 | 6.316E-04 | 7.430E-04 | 1.750E+00 | 2.420E+00 | 1.260E+00 |
| ZNF646 | 5.943E-01 | 2.129E+00 | 2.322E-04 | 4.185E-01 | 4.180E-01 | 8.740E-01 | 1.210E+00 | 6.300E-01 |
| KLF13 | 5.270E-01 | 3.878E+00 | 1.237E-02 | 3.543E-02 | 3.640E-02 | 6.880E-01 | 9.770E-01 | 4.840E-01 |
| ADNP | 7.378E-01 | 3.830E+00 | 3.362E-05 | 2.405E-01 | 2.420E-01 | 8.060E-01 | 1.160E+00 | 5.610E-01 |
| SPI1 | 9.415E-01 | 3.602E+00 | 2.712E-02 | 1.437E-01 | 1.450E-01 | 1.350E+00 | 2.020E+00 | 9.020E-01 |
| ZBTB24 | 5.668E-01 | 1.906E+00 | 9.930E-06 | 3.842E-01 | 3.840E-01 | 8.590E-01 | 1.210E+00 | 6.100E-01 |
| E2F2 | 8.584E-01 | 1.566E+00 | 2.529E-03 | 6.315E-04 | 7.760E-04 | 5.260E-01 | 7.650E-01 | 3.620E-01 |
| ZNF107 | 6.680E-01 | 1.301E+00 | 4.158E-05 | 4.081E-01 | 4.080E-01 | 1.160E+00 | 1.670E+00 | 8.130E-01 |
| ZNF121 | 7.410E-01 | 2.183E+00 | 1.675E-05 | 8.081E-03 | 8.760E-03 | 6.040E-01 | 8.800E-01 | 4.140E-01 |
| RUNX3 | 1.163E+00 | 2.432E+00 | 2.114E-03 | 1.348E-01 | 1.360E-01 | 7.620E-01 | 1.090E+00 | 5.330E-01 |
| BCL11B | 7.089E-01 | 1.512E+00 | 2.791E-03 | 1.579E-03 | 1.780E-03 | 5.940E-01 | 8.240E-01 | 4.290E-01 |
| ETV5 | 6.319E-01 | 2.037E+00 | 2.070E-02 | 1.674E-01 | 1.690E-01 | 1.310E+00 | 1.920E+00 | 8.920E-01 |
| ZNF341 | 4.527E-01 | 1.205E+00 | 9.341E-07 | 1.087E-01 | 1.100E-01 | 7.310E-01 | 1.070E+00 | 4.970E-01 |
| MYB | 1.501E+00 | 1.734E+00 | 1.988E-06 | 1.347E-03 | 1.630E-03 | 5.190E-01 | 7.810E-01 | 3.450E-01 |
| NR2C1 | 5.618E-01 | 1.816E+00 | 2.053E-04 | 2.287E-01 | 2.290E-01 | 7.870E-01 | 1.160E+00 | 5.330E-01 |
| ZNF711 | 4.725E-01 | 1.170E+00 | 5.559E-03 | 1.705E-01 | 1.720E-01 | 7.850E-01 | 1.110E+00 | 5.540E-01 |
| E2F4 | 5.790E-01 | 4.301E+00 | 7.717E-05 | 1.938E-01 | 1.950E-01 | 8.050E-01 | 1.120E+00 | 5.800E-01 |
| KDM5C | 6.106E-01 | 3.659E+00 | 2.192E-03 | 6.107E-02 | 6.220E-02 | 7.070E-01 | 1.020E+00 | 4.900E-01 |
| ZBED1 | 5.365E-01 | 3.730E+00 | 6.354E-03 | 4.641E-02 | 4.740E-02 | 7.060E-01 | 9.960E-01 | 5.010E-01 |
| RUNX1 | 1.254E+00 | 2.200E+00 | 1.141E-06 | 1.535E-01 | 1.550E-01 | 1.270E+00 | 1.760E+00 | 9.150E-01 |
| GATA3 | 8.465E-01 | 1.255E+00 | 2.806E-04 | 1.491E-01 | 1.500E-01 | 1.290E+00 | 1.820E+00 | 9.120E-01 |
| TGIF2 | 1.108E+00 | 2.632E+00 | 6.669E-07 | 1.001E-01 | 1.010E-01 | 1.340E+00 | 1.900E+00 | 9.440E-01 |
| SP140L | 9.987E-01 | 2.090E+00 | 1.481E-07 | 1.062E-01 | 1.070E-01 | 7.490E-01 | 1.060E+00 | 5.270E-01 |
| IRF3 | 8.774E-01 | 3.357E+00 | 1.882E-05 | 1.437E-02 | 1.520E-02 | 6.480E-01 | 9.200E-01 | 4.570E-01 |
| ZNF444 | 3.537E-01 | 2.549E+00 | 4.647E-02 | 6.066E-02 | 6.180E-02 | 1.400E+00 | 1.990E+00 | 9.840E-01 |
| ZBTB6 | 3.488E-01 | 1.992E+00 | 2.544E-03 | 7.473E-02 | 7.570E-02 | 7.310E-01 | 1.030E+00 | 5.170E-01 |
| NCOR2 | 7.138E-01 | 4.435E+00 | 1.174E-03 | 1.633E-01 | 1.640E-01 | 7.670E-01 | 1.110E+00 | 5.280E-01 |
| TFAP2A | 8.916E-01 | 1.444E+00 | 1.887E-02 | 1.647E-02 | 1.750E-02 | 6.330E-01 | 9.230E-01 | 4.340E-01 |
| YBX2 | 1.049E+00 | 1.394E+00 | 2.095E-03 | 2.320E-02 | 2.400E-02 | 6.870E-01 | 9.520E-01 | 4.960E-01 |
| GRHL2 | 9.392E-01 | 2.309E+00 | 8.759E-03 | 4.832E-03 | 5.290E-03 | 6.080E-01 | 8.630E-01 | 4.280E-01 |
| NFE2L3 | 2.203E+00 | 2.855E+00 | 7.267E-12 | 6.586E-04 | 7.900E-04 | 5.520E-01 | 7.810E-01 | 3.900E-01 |
| ZNF207 | 4.593E-01 | 3.234E+00 | 3.043E-04 | 2.574E-02 | 2.680E-02 | 1.470E+00 | 2.080E+00 | 1.050E+00 |
| ZNF367 | 1.206E+00 | 1.763E+00 | 3.179E-08 | 4.063E-03 | 4.540E-03 | 5.790E-01 | 8.450E-01 | 3.970E-01 |
| SNAI1 | 7.649E-01 | 2.011E+00 | 1.149E-02 | 5.407E-03 | 5.860E-03 | 1.610E+00 | 2.260E+00 | 1.150E+00 |
| RBAK | 3.676E-01 | 1.731E+00 | 4.852E-02 | 2.955E-01 | 2.960E-01 | 8.400E-01 | 1.170E+00 | 6.050E-01 |
| TFCP2 | 4.989E-01 | 3.229E+00 | 1.406E-04 | 1.338E-01 | 1.350E-01 | 7.700E-01 | 1.080E+00 | 5.460E-01 |
| ARNTL2 | 9.173E-01 | 2.497E+00 | 9.188E-03 | 2.632E-02 | 2.730E-02 | 6.840E-01 | 9.580E-01 | 4.880E-01 |
| ZNF707 | 6.110E-01 | 1.248E+00 | 7.437E-08 | 1.776E-02 | 1.860E-02 | 6.670E-01 | 9.340E-01 | 4.760E-01 |
| E2F5 | 6.981E-01 | 1.361E+00 | 1.110E-05 | 4.002E-02 | 4.130E-02 | 6.860E-01 | 9.850E-01 | 4.780E-01 |
| ATF6B | 6.025E-01 | 4.847E+00 | 3.436E-04 | 4.182E-02 | 4.280E-02 | 7.140E-01 | 9.890E-01 | 5.150E-01 |
| ELF4 | 7.069E-01 | 3.800E+00 | 1.995E-02 | 1.889E-01 | 1.900E-01 | 7.940E-01 | 1.120E+00 | 5.630E-01 |
| ZBTB2 | 3.872E-01 | 2.811E+00 | 1.940E-02 | 1.835E-01 | 1.840E-01 | 1.250E+00 | 1.730E+00 | 9.000E-01 |
| CDX2 | 1.470E+00 | 2.064E+00 | 1.269E-02 | 1.027E-02 | 1.110E-02 | 6.050E-01 | 8.920E-01 | 4.100E-01 |
| ZNF600 | 4.791E-01 | 1.375E+00 | 2.425E-03 | 1.917E-02 | 2.000E-02 | 6.680E-01 | 9.380E-01 | 4.760E-01 |
| USF1 | 5.663E-01 | 3.920E+00 | 9.278E-04 | 1.753E-03 | 1.970E-03 | 5.980E-01 | 8.280E-01 | 4.320E-01 |
| ZNF74 | 4.721E-01 | 1.522E+00 | 2.356E-03 | 7.828E-02 | 7.940E-02 | 7.410E-01 | 1.040E+00 | 5.310E-01 |
| ZNF213 | 3.232E-01 | 2.474E+00 | 1.161E-02 | 1.475E-01 | 1.490E-01 | 1.290E+00 | 1.810E+00 | 9.140E-01 |
| RFX1 | 5.029E-01 | 2.528E+00 | 8.670E-05 | 3.509E-02 | 3.620E-02 | 6.770E-01 | 9.750E-01 | 4.690E-01 |
| ZNF721 | 3.898E-01 | 1.611E+00 | 3.438E-02 | 4.919E-02 | 5.020E-02 | 7.130E-01 | 1.000E+00 | 5.090E-01 |
| ZNF189 | 5.654E-01 | 2.535E+00 | 2.164E-04 | 3.605E-01 | 3.600E-01 | 8.440E-01 | 1.210E+00 | 5.860E-01 |
| ELK4 | 5.358E-01 | 2.260E+00 | 8.841E-04 | 2.894E-02 | 3.000E-02 | 1.470E+00 | 2.080E+00 | 1.040E+00 |
| SP3 | 3.428E-01 | 2.838E+00 | 3.033E-02 | 2.428E-01 | 2.430E-01 | 8.230E-01 | 1.140E+00 | 5.930E-01 |
| FOXP3 | 1.308E+00 | 1.439E+00 | 2.069E-05 | 7.140E-02 | 7.250E-02 | 7.210E-01 | 1.030E+00 | 5.050E-01 |
| ZBTB46 | 4.688E-01 | 1.216E+00 | 2.207E-03 | 5.508E-01 | 5.500E-01 | 9.050E-01 | 1.250E+00 | 6.540E-01 |
| CERS2 | 7.582E-01 | 4.749E+00 | 1.523E-03 | 1.723E-01 | 1.730E-01 | 7.980E-01 | 1.100E+00 | 5.770E-01 |
| STAT1 | 1.324E+00 | 4.909E+00 | 5.065E-05 | 5.960E-01 | 5.960E-01 | 9.150E-01 | 1.270E+00 | 6.590E-01 |
| BPTF | 3.705E-01 | 2.760E+00 | 2.395E-02 | 1.454E-01 | 1.470E-01 | 7.840E-01 | 1.090E+00 | 5.650E-01 |
| ZNF408 | 5.482E-01 | 2.362E+00 | 1.139E-04 | 1.215E-02 | 1.280E-02 | 6.470E-01 | 9.120E-01 | 4.600E-01 |
| ELF1 | 3.692E-01 | 4.264E+00 | 3.177E-02 | 4.704E-01 | 4.700E-01 | 8.860E-01 | 1.230E+00 | 6.390E-01 |
| ZNF276 | 4.029E-01 | 1.662E+00 | 2.596E-02 | 4.624E-02 | 4.720E-02 | 1.390E+00 | 1.930E+00 | 1.000E+00 |
| PLAGL2 | 7.849E-01 | 3.265E+00 | 1.250E-03 | 1.270E-01 | 1.280E-01 | 7.730E-01 | 1.080E+00 | 5.550E-01 |
| E4F1 | 4.055E-01 | 2.069E+00 | 1.536E-04 | 2.397E-01 | 2.410E-01 | 8.070E-01 | 1.150E+00 | 5.640E-01 |
| HES6 | 1.250E+00 | 2.424E+00 | 1.075E-04 | 8.448E-02 | 8.590E-02 | 7.140E-01 | 1.050E+00 | 4.860E-01 |
| ZKSCAN1 | 5.407E-01 | 3.564E+00 | 1.824E-02 | 1.488E-01 | 1.500E-01 | 7.500E-01 | 1.110E+00 | 5.060E-01 |
| E2F6 | 4.667E-01 | 1.514E+00 | 5.221E-05 | 3.107E-02 | 3.220E-02 | 6.710E-01 | 9.670E-01 | 4.660E-01 |
| ELF3 | 1.366E+00 | 4.781E+00 | 2.874E-02 | 1.299E-01 | 1.310E-01 | 7.640E-01 | 1.080E+00 | 5.390E-01 |
| REPIN1 | 9.176E-01 | 4.999E+00 | 3.251E-05 | 2.684E-04 | 3.280E-04 | 5.480E-01 | 7.610E-01 | 3.950E-01 |
| NFXL1 | 5.992E-01 | 1.559E+00 | 5.049E-06 | 1.375E-01 | 1.390E-01 | 7.630E-01 | 1.090E+00 | 5.330E-01 |
| LZTS1 | 6.642E-01 | 1.212E+00 | 6.775E-03 | 2.961E-02 | 3.060E-02 | 1.460E+00 | 2.040E+00 | 1.040E+00 |
| ZNF165 | 6.759E-01 | 1.400E+00 | 1.079E-03 | 1.093E-01 | 1.110E-01 | 7.660E-01 | 1.060E+00 | 5.520E-01 |
| HIF1A | 5.633E-01 | 4.929E+00 | 1.560E-02 | 4.979E-02 | 5.130E-02 | 1.420E+00 | 2.030E+00 | 9.980E-01 |
| ZNF786 | 3.637E-01 | 1.601E+00 | 9.648E-03 | 2.741E-02 | 2.840E-02 | 6.670E-01 | 9.580E-01 | 4.640E-01 |
| SMAD3 | 4.281E-01 | 3.265E+00 | 4.829E-02 | 3.666E-01 | 3.670E-01 | 1.170E+00 | 1.660E+00 | 8.300E-01 |
| NCOA3 | 5.482E-01 | 3.287E+00 | 1.066E-02 | 2.231E-01 | 2.240E-01 | 1.230E+00 | 1.710E+00 | 8.810E-01 |
| PIAS4 | 3.236E-01 | 3.154E+00 | 4.568E-02 | 7.785E-03 | 8.290E-03 | 6.450E-01 | 8.930E-01 | 4.660E-01 |
| HOXB7 | 1.497E+00 | 3.000E+00 | 3.856E-04 | 1.250E-02 | 1.310E-02 | 6.620E-01 | 9.170E-01 | 4.780E-01 |
| LCORL | 3.573E-01 | 1.294E+00 | 4.659E-02 | 1.577E-02 | 1.660E-02 | 6.420E-01 | 9.230E-01 | 4.470E-01 |
| E2F8 | 1.012E+00 | 1.788E+00 | 1.342E-04 | 9.281E-04 | 1.080E-03 | 5.700E-01 | 7.990E-01 | 4.070E-01 |
| SETDB1 | 6.117E-01 | 2.351E+00 | 1.827E-07 | 1.757E-01 | 1.770E-01 | 7.990E-01 | 1.110E+00 | 5.770E-01 |
| PAX8 | 8.387E-01 | 1.002E+00 | 1.114E-04 | 5.062E-01 | 5.060E-01 | 8.870E-01 | 1.260E+00 | 6.240E-01 |
| ZNF343 | 3.991E-01 | 1.479E+00 | 6.360E-03 | 1.640E-01 | 1.650E-01 | 7.690E-01 | 1.110E+00 | 5.310E-01 |
| TCF3 | 1.175E+00 | 3.655E+00 | 1.014E-07 | 1.157E-02 | 1.230E-02 | 6.320E-01 | 9.050E-01 | 4.420E-01 |
| ZNF628 | 3.986E-01 | 2.254E+00 | 2.119E-03 | 1.917E-01 | 1.930E-01 | 7.940E-01 | 1.120E+00 | 5.600E-01 |
| PHF20 | 4.479E-01 | 2.667E+00 | 2.105E-03 | 1.320E-01 | 1.330E-01 | 7.380E-01 | 1.100E+00 | 4.960E-01 |
| ZFP41 | 5.541E-01 | 1.285E+00 | 7.211E-06 | 9.078E-02 | 9.180E-02 | 7.530E-01 | 1.050E+00 | 5.410E-01 |
| ZNF3 | 4.565E-01 | 2.514E+00 | 3.168E-03 | 1.219E-02 | 1.290E-02 | 6.550E-01 | 9.140E-01 | 4.690E-01 |
| ZBED4 | 8.467E-01 | 2.453E+00 | 1.710E-06 | 1.396E-01 | 1.410E-01 | 7.590E-01 | 1.100E+00 | 5.250E-01 |
| ZNF644 | 3.669E-01 | 2.469E+00 | 9.140E-03 | 3.716E-01 | 3.710E-01 | 8.550E-01 | 1.210E+00 | 6.050E-01 |
| MSX2 | 9.972E-01 | 1.798E+00 | 7.082E-03 | 7.355E-03 | 7.890E-03 | 6.210E-01 | 8.820E-01 | 4.360E-01 |
| ZNF335 | 6.006E-01 | 2.404E+00 | 3.249E-05 | 4.661E-01 | 4.670E-01 | 1.130E+00 | 1.580E+00 | 8.110E-01 |
| SMAD6 | 8.715E-01 | 1.830E+00 | 3.991E-04 | 2.141E-03 | 2.370E-03 | 6.040E-01 | 8.360E-01 | 4.360E-01 |
| ZNF92 | 6.581E-01 | 1.912E+00 | 8.853E-03 | 1.297E-01 | 1.310E-01 | 7.780E-01 | 1.080E+00 | 5.610E-01 |
| HNF1A | 1.264E+00 | 1.694E+00 | 7.860E-05 | 1.122E-01 | 1.140E-01 | 7.470E-01 | 1.070E+00 | 5.200E-01 |
| ZMIZ2 | 1.085E+00 | 3.959E+00 | 1.462E-08 | 2.367E-01 | 2.380E-01 | 8.140E-01 | 1.150E+00 | 5.780E-01 |
| ZNF749 | 3.651E-01 | 1.161E+00 | 3.700E-03 | 1.846E-01 | 1.850E-01 | 8.020E-01 | 1.110E+00 | 5.790E-01 |
| RCOR2 | 9.813E-01 | 1.167E+00 | 8.128E-05 | 2.293E-01 | 2.300E-01 | 8.130E-01 | 1.140E+00 | 5.800E-01 |
| FARSA | 8.225E-01 | 4.431E+00 | 8.427E-06 | 1.499E-03 | 1.700E-03 | 5.890E-01 | 8.200E-01 | 4.230E-01 |
| BATF2 | 1.287E+00 | 2.252E+00 | 2.171E-03 | 3.639E-03 | 3.980E-03 | 6.090E-01 | 8.540E-01 | 4.350E-01 |
| AHR | 9.092E-01 | 4.430E+00 | 1.101E-06 | 2.038E-01 | 2.050E-01 | 1.260E+00 | 1.790E+00 | 8.830E-01 |
| ASCL2 | 2.182E+00 | 2.568E+00 | 3.627E-04 | 1.176E-02 | 1.260E-02 | 6.130E-01 | 9.010E-01 | 4.170E-01 |
| HOXB13 | 1.394E+00 | 1.458E+00 | 1.534E-03 | 1.217E-02 | 1.290E-02 | 6.360E-01 | 9.090E-01 | 4.460E-01 |
| ZNF587 | 6.198E-01 | 1.981E+00 | 2.117E-04 | 2.238E-01 | 2.240E-01 | 8.010E-01 | 1.150E+00 | 5.610E-01 |
| MSC | 1.021E+00 | 2.106E+00 | 7.304E-04 | 1.838E-02 | 1.940E-02 | 1.580E+00 | 2.330E+00 | 1.080E+00 |
| KDM5A | 3.957E-01 | 2.480E+00 | 1.198E-03 | 1.914E-01 | 1.920E-01 | 8.020E-01 | 1.120E+00 | 5.750E-01 |
| BAZ1A | 8.020E-01 | 3.101E+00 | 2.735E-05 | 2.990E-01 | 3.000E-01 | 8.390E-01 | 1.170E+00 | 6.010E-01 |
| HNF4G | 1.325E+00 | 2.523E+00 | 2.271E-02 | 2.525E-02 | 2.660E-02 | 6.310E-01 | 9.480E-01 | 4.200E-01 |
| SP1 | 3.875E-01 | 4.193E+00 | 3.152E-02 | 4.439E-02 | 4.570E-02 | 6.720E-01 | 9.920E-01 | 4.550E-01 |
| HEYL | 8.993E-01 | 2.343E+00 | 1.016E-02 | 6.491E-04 | 7.670E-04 | 1.780E+00 | 2.490E+00 | 1.270E+00 |
| ZNF48 | 4.516E-01 | 1.886E+00 | 2.332E-02 | 8.453E-02 | 8.600E-02 | 7.320E-01 | 1.050E+00 | 5.120E-01 |
| ZNF131 | 6.804E-01 | 1.969E+00 | 1.439E-05 | 1.316E-02 | 1.380E-02 | 6.620E-01 | 9.190E-01 | 4.770E-01 |
| ZSCAN25 | 3.315E-01 | 1.526E+00 | 1.958E-02 | 6.772E-02 | 6.880E-02 | 7.300E-01 | 1.020E+00 | 5.200E-01 |
| ZBTB41 | 4.116E-01 | 1.599E+00 | 4.328E-03 | 2.776E-01 | 2.780E-01 | 8.230E-01 | 1.170E+00 | 5.790E-01 |
| PDX1 | 1.380E+00 | 2.273E+00 | 5.370E-03 | 3.858E-02 | 3.960E-02 | 7.110E-01 | 9.840E-01 | 5.130E-01 |
| HES4 | 7.306E-01 | 3.510E+00 | 1.862E-02 | 4.938E-01 | 4.940E-01 | 8.800E-01 | 1.270E+00 | 6.090E-01 |
| ZNF562 | 3.324E-01 | 1.869E+00 | 3.663E-02 | 2.061E-03 | 2.290E-03 | 6.030E-01 | 8.350E-01 | 4.350E-01 |
| TEAD4 | 1.426E+00 | 2.730E+00 | 7.879E-10 | 8.464E-03 | 9.290E-03 | 5.820E-01 | 8.750E-01 | 3.880E-01 |
| MLXIPL | 1.100E+00 | 1.488E+00 | 1.184E-02 | 3.448E-01 | 3.460E-01 | 8.340E-01 | 1.220E+00 | 5.720E-01 |
| ZNF267 | 7.799E-01 | 1.587E+00 | 4.323E-06 | 2.865E-01 | 2.880E-01 | 1.220E+00 | 1.780E+00 | 8.430E-01 |
| GATAD2A | 7.744E-01 | 3.600E+00 | 5.930E-06 | 4.275E-02 | 4.380E-02 | 6.990E-01 | 9.900E-01 | 4.930E-01 |
| CREBZF | 6.251E-01 | 2.735E+00 | 1.676E-03 | 2.630E-02 | 2.710E-02 | 6.910E-01 | 9.590E-01 | 4.970E-01 |
| ETV4 | 2.384E+00 | 2.455E+00 | 1.277E-09 | 2.016E-01 | 2.020E-01 | 8.080E-01 | 1.120E+00 | 5.810E-01 |
| APEX2 | 7.800E-01 | 3.484E+00 | 4.757E-05 | 3.913E-02 | 4.020E-02 | 6.890E-01 | 9.830E-01 | 4.820E-01 |
| SNAPC4 | 7.921E-01 | 2.097E+00 | 2.095E-08 | 1.720E-02 | 1.790E-02 | 6.750E-01 | 9.350E-01 | 4.880E-01 |
| FOXK1 | 5.354E-01 | 2.698E+00 | 2.201E-02 | 1.167E-01 | 1.180E-01 | 7.620E-01 | 1.070E+00 | 5.420E-01 |
| ZNF138 | 4.787E-01 | 1.747E+00 | 5.839E-04 | 2.861E-01 | 2.860E-01 | 1.210E+00 | 1.700E+00 | 8.550E-01 |
| ZNF618 | 7.419E-01 | 1.564E+00 | 2.083E-04 | 6.664E-02 | 6.770E-02 | 7.320E-01 | 1.020E+00 | 5.230E-01 |
| ZNF184 | 4.050E-01 | 1.763E+00 | 6.616E-03 | 1.647E-01 | 1.660E-01 | 7.900E-01 | 1.100E+00 | 5.660E-01 |
| ETV6 | 7.528E-01 | 3.268E+00 | 2.110E-06 | 1.561E-02 | 1.630E-02 | 6.700E-01 | 9.290E-01 | 4.840E-01 |
| ZNF281 | 6.971E-01 | 2.148E+00 | 2.865E-07 | 2.091E-02 | 2.180E-02 | 1.480E+00 | 2.070E+00 | 1.060E+00 |
| ZNF696 | 6.046E-01 | 1.404E+00 | 1.905E-07 | 1.131E-01 | 1.140E-01 | 7.560E-01 | 1.070E+00 | 5.330E-01 |
| E2F1 | 1.953E+00 | 2.628E+00 | 8.265E-11 | 1.534E-02 | 1.610E-02 | 6.610E-01 | 9.260E-01 | 4.710E-01 |
| SREBF1 | 7.798E-01 | 3.721E+00 | 6.151E-04 | 1.121E-02 | 1.190E-02 | 6.280E-01 | 9.030E-01 | 4.370E-01 |
| MBD6 | 4.701E-01 | 3.914E+00 | 7.992E-03 | 1.957E-01 | 1.970E-01 | 1.250E+00 | 1.750E+00 | 8.910E-01 |
| FOXP4 | 1.070E+00 | 4.673E+00 | 5.236E-05 | 3.860E-02 | 3.980E-02 | 6.810E-01 | 9.820E-01 | 4.730E-01 |
| NFKB2 | 1.185E+00 | 4.078E+00 | 8.713E-06 | 2.287E-03 | 2.530E-03 | 5.980E-01 | 8.350E-01 | 4.280E-01 |
| ZNF764 | 3.247E-01 | 2.086E+00 | 3.337E-02 | 3.304E-01 | 3.310E-01 | 1.180E+00 | 1.630E+00 | 8.490E-01 |
| E2F3 | 1.081E+00 | 1.872E+00 | 2.909E-10 | 2.990E-01 | 2.990E-01 | 1.200E+00 | 1.710E+00 | 8.480E-01 |
| ADAR | 9.273E-01 | 5.104E+00 | 3.484E-07 | 3.664E-01 | 3.670E-01 | 8.460E-01 | 1.220E+00 | 5.870E-01 |
| AHCTF1 | 4.899E-01 | 2.465E+00 | 4.050E-03 | 1.194E-01 | 1.210E-01 | 1.290E+00 | 1.790E+00 | 9.350E-01 |
| AKAP8 | 4.096E-01 | 2.891E+00 | 1.960E-04 | 1.222E-02 | 1.290E-02 | 6.480E-01 | 9.120E-01 | 4.600E-01 |
| AKNA | 6.793E-01 | 2.706E+00 | 3.971E-02 | 2.817E-02 | 2.920E-02 | 6.800E-01 | 9.620E-01 | 4.810E-01 |
| ANAPC2 | 5.143E-01 | 2.874E+00 | 3.754E-05 | 1.091E-03 | 1.280E-03 | 5.580E-01 | 7.960E-01 | 3.920E-01 |
| ANKZF1 | 5.409E-01 | 2.111E+00 | 1.140E-04 | 1.233E-02 | 1.310E-02 | 6.480E-01 | 9.130E-01 | 4.600E-01 |
| APTX | 4.867E-01 | 2.222E+00 | 1.431E-05 | 9.917E-02 | 1.000E-01 | 7.500E-01 | 1.060E+00 | 5.310E-01 |
| ARID3A | 7.643E-01 | 1.762E+00 | 1.617E-04 | 3.549E-01 | 3.550E-01 | 1.190E+00 | 1.740E+00 | 8.200E-01 |
| BPNT1 | 5.671E-01 | 3.452E+00 | 4.097E-02 | 1.786E-02 | 1.880E-02 | 6.540E-01 | 9.320E-01 | 4.590E-01 |
| BRD9 | 6.698E-01 | 1.934E+00 | 7.946E-09 | 6.613E-02 | 6.730E-02 | 7.380E-01 | 1.020E+00 | 5.340E-01 |
| BRPF1 | 4.798E-01 | 2.522E+00 | 1.653E-04 | 6.599E-02 | 6.730E-02 | 7.030E-01 | 1.030E+00 | 4.820E-01 |
| CBFB | 1.015E+00 | 3.183E+00 | 7.282E-08 | 1.683E-01 | 1.690E-01 | 1.260E+00 | 1.740E+00 | 9.070E-01 |
| CEBPZ | 3.789E-01 | 3.322E+00 | 6.199E-03 | 3.635E-02 | 3.720E-02 | 7.070E-01 | 9.800E-01 | 5.100E-01 |
| CENPB | 5.255E-01 | 5.496E+00 | 1.825E-02 | 1.673E-01 | 1.680E-01 | 1.280E+00 | 1.810E+00 | 9.020E-01 |
| CHD1 | 3.778E-01 | 2.205E+00 | 1.612E-02 | 3.317E-01 | 3.320E-01 | 8.400E-01 | 1.200E+00 | 5.900E-01 |
| CHD7 | 8.915E-01 | 1.669E+00 | 2.725E-09 | 1.211E-01 | 1.220E-01 | 7.710E-01 | 1.070E+00 | 5.550E-01 |
| CHRAC1 | 5.390E-01 | 3.970E+00 | 1.029E-04 | 1.013E-01 | 1.030E-01 | 7.350E-01 | 1.060E+00 | 5.080E-01 |
| CPSF4 | 5.511E-01 | 2.258E+00 | 2.131E-04 | 2.857E-02 | 2.970E-02 | 6.720E-01 | 9.620E-01 | 4.690E-01 |
| CREB3L1 | 1.467E+00 | 4.848E+00 | 3.319E-02 | 4.572E-01 | 4.590E-01 | 8.680E-01 | 1.260E+00 | 5.980E-01 |
| CUL2 | 3.486E-01 | 2.809E+00 | 5.185E-03 | 2.919E-02 | 3.000E-02 | 6.940E-01 | 9.650E-01 | 4.990E-01 |
| CUL4A | 5.100E-01 | 3.262E+00 | 1.093E-04 | 4.878E-04 | 6.000E-04 | 5.270E-01 | 7.600E-01 | 3.660E-01 |
| DEK | 6.322E-01 | 4.889E+00 | 4.561E-05 | 1.511E-01 | 1.520E-01 | 7.780E-01 | 1.100E+00 | 5.520E-01 |
| DEPDC1 | 1.236E+00 | 1.398E+00 | 1.598E-06 | 1.642E-02 | 1.710E-02 | 6.720E-01 | 9.320E-01 | 4.850E-01 |
| DEPDC1B | 1.170E+00 | 1.476E+00 | 3.975E-07 | 1.097E-01 | 1.110E-01 | 7.670E-01 | 1.060E+00 | 5.540E-01 |
| DHX34 | 9.941E-01 | 2.411E+00 | 1.545E-08 | 9.977E-02 | 1.010E-01 | 7.600E-01 | 1.050E+00 | 5.480E-01 |
| DNAJC2 | 8.064E-01 | 2.091E+00 | 1.259E-07 | 8.447E-02 | 8.560E-02 | 7.400E-01 | 1.040E+00 | 5.240E-01 |
| DOT1L | 6.733E-01 | 2.672E+00 | 1.841E-03 | 2.966E-03 | 3.280E-03 | 6.060E-01 | 8.460E-01 | 4.340E-01 |
| DUS3L | 6.066E-01 | 2.600E+00 | 1.940E-05 | 1.004E-01 | 1.020E-01 | 7.430E-01 | 1.060E+00 | 5.200E-01 |
| DUSP12 | 4.926E-01 | 2.708E+00 | 6.290E-04 | 1.096E-01 | 1.110E-01 | 7.670E-01 | 1.060E+00 | 5.540E-01 |
| EP400 | 4.441E-01 | 2.179E+00 | 1.756E-02 | 2.845E-01 | 2.850E-01 | 1.210E+00 | 1.720E+00 | 8.530E-01 |
| EWSR1 | 5.597E-01 | 4.231E+00 | 4.822E-06 | 7.099E-03 | 7.540E-03 | 6.410E-01 | 8.880E-01 | 4.630E-01 |
| EXOC2 | 3.368E-01 | 2.704E+00 | 5.999E-03 | 1.669E-01 | 1.680E-01 | 1.260E+00 | 1.760E+00 | 9.060E-01 |
| EZH2 | 1.216E+00 | 1.924E+00 | 1.664E-07 | 5.894E-04 | 6.960E-04 | 5.690E-01 | 7.890E-01 | 4.110E-01 |
| FARSB | 5.469E-01 | 3.742E+00 | 1.733E-04 | 2.671E-01 | 2.680E-01 | 1.230E+00 | 1.780E+00 | 8.520E-01 |
| FMNL2 | 8.255E-01 | 2.405E+00 | 1.523E-04 | 1.251E-01 | 1.260E-01 | 1.290E+00 | 1.790E+00 | 9.310E-01 |
| GON4L | 3.985E-01 | 1.928E+00 | 4.381E-03 | 3.305E-01 | 3.310E-01 | 8.500E-01 | 1.180E+00 | 6.120E-01 |
| GTF2F2 | 4.391E-01 | 2.695E+00 | 5.563E-04 | 1.795E-01 | 1.800E-01 | 8.010E-01 | 1.110E+00 | 5.780E-01 |
| HIST1H1E | 7.739E-01 | 2.453E+00 | 2.377E-02 | 7.290E-03 | 7.790E-03 | 6.350E-01 | 8.870E-01 | 4.550E-01 |
| HMG20A | 4.044E-01 | 1.952E+00 | 2.578E-03 | 4.109E-01 | 4.110E-01 | 1.150E+00 | 1.590E+00 | 8.280E-01 |
| HMGA1 | 1.839E+00 | 6.923E+00 | 3.027E-04 | 3.504E-03 | 3.840E-03 | 6.140E-01 | 8.550E-01 | 4.410E-01 |
| HMGB1 | 5.440E-01 | 4.812E+00 | 1.140E-04 | 8.350E-03 | 8.860E-03 | 6.390E-01 | 8.940E-01 | 4.570E-01 |
| HMGB2 | 9.045E-01 | 4.706E+00 | 1.237E-05 | 1.377E-02 | 1.450E-02 | 6.640E-01 | 9.220E-01 | 4.790E-01 |
| HMGB3 | 1.790E+00 | 2.745E+00 | 1.064E-08 | 2.364E-02 | 2.440E-02 | 6.880E-01 | 9.530E-01 | 4.960E-01 |
| HNF1B | 1.255E+00 | 1.730E+00 | 5.846E-04 | 9.571E-02 | 9.660E-02 | 7.500E-01 | 1.050E+00 | 5.340E-01 |
| HSF1 | 6.288E-01 | 4.359E+00 | 5.487E-05 | 1.107E-02 | 1.170E-02 | 6.580E-01 | 9.110E-01 | 4.760E-01 |
| INF2 | 7.871E-01 | 4.213E+00 | 3.707E-03 | 4.697E-01 | 4.700E-01 | 8.760E-01 | 1.250E+00 | 6.120E-01 |
| JRKL | 7.293E-01 | 1.746E+00 | 5.536E-07 | 7.955E-02 | 8.110E-02 | 6.990E-01 | 1.050E+00 | 4.680E-01 |
| KIN | 4.359E-01 | 1.257E+00 | 1.564E-05 | 4.807E-02 | 4.940E-02 | 1.440E+00 | 2.070E+00 | 1.000E+00 |
| KNTC1 | 9.375E-01 | 1.410E+00 | 3.415E-07 | 2.057E-02 | 2.150E-02 | 6.580E-01 | 9.400E-01 | 4.600E-01 |
| LARP1 | 6.600E-01 | 4.496E+00 | 1.087E-04 | 3.323E-01 | 3.330E-01 | 1.180E+00 | 1.630E+00 | 8.480E-01 |
| LARP4 | 6.118E-01 | 2.623E+00 | 8.477E-05 | 2.722E-02 | 2.800E-02 | 6.920E-01 | 9.610E-01 | 4.980E-01 |
| LEF1 | 7.580E-01 | 1.429E+00 | 7.862E-04 | 9.401E-02 | 9.530E-02 | 1.360E+00 | 1.960E+00 | 9.470E-01 |
| MET | 1.600E+00 | 3.074E+00 | 3.681E-07 | 6.186E-02 | 6.340E-02 | 1.470E+00 | 2.210E+00 | 9.790E-01 |
| MIER2 | 4.893E-01 | 2.725E+00 | 3.420E-03 | 1.057E-01 | 1.070E-01 | 7.630E-01 | 1.060E+00 | 5.490E-01 |
| MRRF | 4.324E-01 | 1.924E+00 | 4.610E-04 | 3.261E-02 | 3.360E-02 | 6.840E-01 | 9.710E-01 | 4.820E-01 |
| MST1R | 1.091E+00 | 3.100E+00 | 1.963E-02 | 3.358E-01 | 3.370E-01 | 8.460E-01 | 1.190E+00 | 6.020E-01 |
| MXD3 | 4.883E-01 | 1.028E+00 | 1.009E-03 | 1.276E-02 | 1.350E-02 | 6.420E-01 | 9.130E-01 | 4.520E-01 |
| NFRKB | 4.585E-01 | 2.624E+00 | 3.702E-03 | 7.203E-02 | 7.340E-02 | 7.110E-01 | 1.030E+00 | 4.890E-01 |
| NKRF | 7.487E-01 | 1.570E+00 | 3.003E-07 | 5.065E-02 | 5.160E-02 | 7.220E-01 | 1.000E+00 | 5.200E-01 |
| NOC3L | 6.425E-01 | 2.001E+00 | 3.374E-04 | 3.859E-02 | 3.950E-02 | 7.070E-01 | 9.840E-01 | 5.090E-01 |
| NOC4L | 8.794E-01 | 3.687E+00 | 3.952E-08 | 1.538E-01 | 1.550E-01 | 7.790E-01 | 1.100E+00 | 5.530E-01 |
| NUPL2 | 4.612E-01 | 2.291E+00 | 1.297E-03 | 1.230E-01 | 1.240E-01 | 7.650E-01 | 1.080E+00 | 5.440E-01 |
| ONECUT2 | 1.615E+00 | 1.565E+00 | 2.943E-04 | 3.141E-02 | 3.240E-02 | 1.470E+00 | 2.090E+00 | 1.030E+00 |
| PARP12 | 9.049E-01 | 3.060E+00 | 1.387E-03 | 5.990E-02 | 6.120E-02 | 7.130E-01 | 1.020E+00 | 5.000E-01 |
| PAWR | 5.819E-01 | 2.210E+00 | 3.180E-03 | 4.235E-01 | 4.240E-01 | 1.150E+00 | 1.620E+00 | 8.160E-01 |
| PLEK | 9.526E-01 | 2.473E+00 | 3.820E-02 | 5.731E-01 | 5.730E-01 | 9.110E-01 | 1.260E+00 | 6.580E-01 |
| PLEK2 | 1.355E+00 | 3.711E+00 | 2.428E-02 | 2.684E-01 | 2.690E-01 | 8.290E-01 | 1.160E+00 | 5.940E-01 |
| PLXNA1 | 1.150E+00 | 2.836E+00 | 4.607E-05 | 6.003E-03 | 6.620E-03 | 5.790E-01 | 8.590E-01 | 3.910E-01 |
| PLXNA3 | 8.876E-01 | 2.213E+00 | 1.377E-04 | 4.086E-01 | 4.100E-01 | 8.560E-01 | 1.240E+00 | 5.910E-01 |
| PLXNB2 | 6.475E-01 | 6.010E+00 | 8.272E-03 | 8.080E-04 | 9.390E-04 | 5.730E-01 | 7.970E-01 | 4.120E-01 |
| PLXNC1 | 4.553E-01 | 1.431E+00 | 3.535E-02 | 7.427E-03 | 8.110E-03 | 1.660E+00 | 2.420E+00 | 1.140E+00 |
| PLXND1 | 1.046E+00 | 3.297E+00 | 2.457E-04 | 1.190E-01 | 1.210E-01 | 1.380E+00 | 2.060E+00 | 9.200E-01 |
| PMS1 | 3.687E-01 | 1.600E+00 | 1.136E-02 | 2.723E-01 | 2.730E-01 | 1.210E+00 | 1.690E+00 | 8.620E-01 |
| POGK | 5.908E-01 | 2.971E+00 | 7.909E-05 | 6.013E-01 | 6.020E-01 | 9.170E-01 | 1.270E+00 | 6.610E-01 |
| POLE3 | 7.196E-01 | 4.325E+00 | 4.574E-07 | 2.490E-02 | 2.580E-02 | 6.730E-01 | 9.530E-01 | 4.750E-01 |
| PPP1R10 | 5.009E-01 | 4.004E+00 | 4.146E-04 | 1.242E-01 | 1.260E-01 | 7.600E-01 | 1.080E+00 | 5.350E-01 |
| PREB | 5.878E-01 | 3.909E+00 | 7.286E-03 | 9.644E-02 | 9.750E-02 | 7.490E-01 | 1.050E+00 | 5.330E-01 |
| PRKRIR | 6.298E-01 | 2.486E+00 | 1.013E-03 | 2.648E-01 | 2.660E-01 | 8.120E-01 | 1.170E+00 | 5.630E-01 |
| PRMT3 | 6.023E-01 | 1.749E+00 | 2.356E-05 | 1.431E-01 | 1.450E-01 | 7.540E-01 | 1.100E+00 | 5.150E-01 |
| PRR12 | 6.457E-01 | 3.494E+00 | 1.862E-03 | 2.533E-02 | 2.620E-02 | 6.860E-01 | 9.560E-01 | 4.920E-01 |
| PRR3 | 3.913E-01 | 1.715E+00 | 3.582E-03 | 4.180E-01 | 4.180E-01 | 1.170E+00 | 1.690E+00 | 8.050E-01 |
| PSMD11 | 8.258E-01 | 3.847E+00 | 1.482E-07 | 1.148E-02 | 1.210E-02 | 6.560E-01 | 9.120E-01 | 4.720E-01 |
| PSMD12 | 5.990E-01 | 3.069E+00 | 5.832E-07 | 1.496E-02 | 1.560E-02 | 6.680E-01 | 9.270E-01 | 4.820E-01 |
| RAD51 | 1.289E+00 | 1.623E+00 | 7.879E-10 | 2.124E-02 | 2.200E-02 | 6.830E-01 | 9.470E-01 | 4.920E-01 |
| RAPGEF5 | 4.530E-01 | 1.841E+00 | 1.531E-02 | 7.993E-03 | 8.570E-03 | 1.620E+00 | 2.320E+00 | 1.130E+00 |
| RBM10 | 5.210E-01 | 3.975E+00 | 1.835E-04 | 3.918E-02 | 4.010E-02 | 7.110E-01 | 9.850E-01 | 5.130E-01 |
| RBM22 | 3.260E-01 | 3.760E+00 | 2.885E-03 | 1.690E-01 | 1.700E-01 | 7.850E-01 | 1.110E+00 | 5.560E-01 |
| RBM26 | 5.069E-01 | 2.944E+00 | 2.943E-03 | 8.829E-02 | 8.940E-02 | 7.370E-01 | 1.050E+00 | 5.180E-01 |
| RNF113A | 4.712E-01 | 3.196E+00 | 3.010E-03 | 7.093E-02 | 7.200E-02 | 7.380E-01 | 1.030E+00 | 5.310E-01 |
| RNF114 | 9.161E-01 | 4.089E+00 | 7.435E-07 | 1.777E-01 | 1.790E-01 | 7.720E-01 | 1.130E+00 | 5.300E-01 |
| RNF138 | 3.987E-01 | 2.892E+00 | 1.565E-03 | 2.224E-02 | 2.300E-02 | 6.780E-01 | 9.480E-01 | 4.850E-01 |
| RNF166 | 5.381E-01 | 2.218E+00 | 1.029E-02 | 1.374E-01 | 1.380E-01 | 1.280E+00 | 1.770E+00 | 9.240E-01 |
| RPA2 | 4.448E-01 | 3.774E+00 | 8.910E-03 | 7.862E-02 | 8.020E-02 | 7.010E-01 | 1.040E+00 | 4.720E-01 |
| SF3A2 | 7.095E-01 | 5.117E+00 | 3.195E-06 | 6.082E-03 | 6.580E-03 | 6.190E-01 | 8.750E-01 | 4.380E-01 |
| SF3A3 | 5.497E-01 | 3.635E+00 | 3.004E-07 | 1.097E-02 | 1.150E-02 | 6.560E-01 | 9.100E-01 | 4.730E-01 |
| SKIL | 6.396E-01 | 3.029E+00 | 3.113E-03 | 2.609E-02 | 2.710E-02 | 1.460E+00 | 2.050E+00 | 1.040E+00 |
| SLC39A10 | 1.068E+00 | 1.653E+00 | 4.829E-07 | 1.885E-02 | 1.970E-02 | 1.530E+00 | 2.180E+00 | 1.070E+00 |
| SMARCA5 | 5.247E-01 | 3.357E+00 | 4.338E-03 | 2.347E-01 | 2.350E-01 | 8.160E-01 | 1.140E+00 | 5.830E-01 |
| SOX12 | 7.516E-01 | 2.575E+00 | 4.926E-03 | 9.399E-02 | 9.550E-02 | 1.380E+00 | 2.020E+00 | 9.450E-01 |
| SOX13 | 5.558E-01 | 2.386E+00 | 4.273E-03 | 1.438E-01 | 1.450E-01 | 7.760E-01 | 1.090E+00 | 5.520E-01 |
| SOX4 | 1.921E+00 | 3.850E+00 | 6.671E-10 | 5.344E-02 | 5.440E-02 | 1.380E+00 | 1.920E+00 | 9.940E-01 |
| SOX9 | 2.526E+00 | 4.604E+00 | 7.518E-07 | 4.493E-03 | 4.860E-03 | 6.220E-01 | 8.660E-01 | 4.470E-01 |
| SRCAP | 6.483E-01 | 3.725E+00 | 2.331E-04 | 2.963E-01 | 2.970E-01 | 8.250E-01 | 1.180E+00 | 5.740E-01 |
| SSB | 6.723E-01 | 3.368E+00 | 1.838E-04 | 1.701E-01 | 1.710E-01 | 7.830E-01 | 1.110E+00 | 5.510E-01 |
| SSH2 | 5.567E-01 | 1.758E+00 | 2.937E-05 | 1.023E-01 | 1.030E-01 | 7.610E-01 | 1.060E+00 | 5.480E-01 |
| SSRP1 | 8.144E-01 | 4.230E+00 | 5.464E-08 | 3.728E-02 | 3.830E-02 | 6.880E-01 | 9.800E-01 | 4.830E-01 |
| SUZ12 | 7.728E-01 | 2.873E+00 | 1.339E-07 | 2.065E-01 | 2.080E-01 | 8.040E-01 | 1.130E+00 | 5.730E-01 |
| TCF19 | 1.041E+00 | 2.790E+00 | 1.080E-05 | 2.151E-02 | 2.240E-02 | 6.720E-01 | 9.450E-01 | 4.780E-01 |
| TCF20 | 7.839E-01 | 3.211E+00 | 3.855E-05 | 3.089E-03 | 3.480E-03 | 5.700E-01 | 8.310E-01 | 3.910E-01 |
| TCF7 | 1.118E+00 | 2.087E+00 | 8.825E-04 | 1.730E-02 | 1.800E-02 | 6.640E-01 | 9.320E-01 | 4.730E-01 |
| TFAM | 6.792E-01 | 2.755E+00 | 8.335E-06 | 1.505E-01 | 1.520E-01 | 7.750E-01 | 1.100E+00 | 5.470E-01 |
| TIGD2 | 4.888E-01 | 1.707E+00 | 5.714E-03 | 3.157E-01 | 3.160E-01 | 1.180E+00 | 1.640E+00 | 8.520E-01 |
| TIGD5 | 6.065E-01 | 1.838E+00 | 3.698E-06 | 2.058E-02 | 2.140E-02 | 6.770E-01 | 9.440E-01 | 4.850E-01 |
| TOE1 | 4.936E-01 | 2.538E+00 | 7.543E-04 | 7.089E-04 | 8.260E-04 | 5.740E-01 | 7.950E-01 | 4.150E-01 |
| TRIM32 | 3.377E-01 | 2.236E+00 | 1.337E-02 | 7.041E-02 | 7.160E-02 | 1.370E+00 | 1.920E+00 | 9.730E-01 |
| TRIT1 | 3.777E-01 | 2.213E+00 | 6.122E-03 | 4.515E-02 | 4.620E-02 | 7.170E-01 | 9.940E-01 | 5.170E-01 |
| TRMT1 | 7.687E-01 | 2.753E+00 | 2.978E-07 | 1.769E-03 | 1.990E-03 | 5.980E-01 | 8.280E-01 | 4.320E-01 |
| TTF1 | 3.949E-01 | 2.116E+00 | 4.838E-03 | 9.556E-03 | 1.010E-02 | 6.510E-01 | 9.030E-01 | 4.700E-01 |
| TUT1 | 4.902E-01 | 1.372E+00 | 4.429E-06 | 2.001E-02 | 2.100E-02 | 6.610E-01 | 9.390E-01 | 4.650E-01 |
| UBTF | 3.708E-01 | 3.867E+00 | 7.412E-03 | 7.591E-02 | 7.690E-02 | 7.450E-01 | 1.030E+00 | 5.370E-01 |
| USP39 | 5.463E-01 | 3.541E+00 | 2.735E-07 | 3.930E-03 | 4.270E-03 | 6.230E-01 | 8.620E-01 | 4.500E-01 |
| WDHD1 | 8.000E-01 | 1.174E+00 | 7.437E-08 | 7.974E-03 | 8.490E-03 | 6.460E-01 | 8.940E-01 | 4.660E-01 |
| WHSC1 | 9.882E-01 | 2.111E+00 | 1.969E-06 | 3.807E-02 | 3.910E-02 | 6.980E-01 | 9.820E-01 | 4.970E-01 |
| YEATS2 | 5.266E-01 | 2.100E+00 | 3.516E-03 | 2.790E-01 | 2.800E-01 | 8.310E-01 | 1.160E+00 | 5.940E-01 |
| YEATS4 | 7.167E-01 | 3.062E+00 | 5.448E-05 | 1.326E-01 | 1.340E-01 | 7.610E-01 | 1.090E+00 | 5.330E-01 |
| ZC3H11A | 4.341E-01 | 3.461E+00 | 2.190E-02 | 2.608E-01 | 2.610E-01 | 8.260E-01 | 1.150E+00 | 5.910E-01 |
| ZC3H15 | 4.738E-01 | 4.042E+00 | 4.877E-03 | 2.214E-01 | 2.220E-01 | 8.120E-01 | 1.130E+00 | 5.820E-01 |
| ZC3H18 | 6.511E-01 | 2.639E+00 | 4.403E-06 | 8.424E-02 | 8.550E-02 | 7.330E-01 | 1.040E+00 | 5.150E-01 |
| ZC3H3 | 9.649E-01 | 3.546E+00 | 3.496E-09 | 5.333E-02 | 5.430E-02 | 7.260E-01 | 1.010E+00 | 5.240E-01 |
| ZC3H4 | 5.468E-01 | 3.317E+00 | 2.149E-05 | 5.566E-03 | 6.040E-03 | 6.120E-01 | 8.690E-01 | 4.320E-01 |
| ZC3H7B | 4.763E-01 | 3.502E+00 | 4.723E-03 | 3.308E-02 | 3.390E-02 | 7.030E-01 | 9.740E-01 | 5.080E-01 |
| ZC3H8 | 3.320E-01 | 1.140E+00 | 8.067E-03 | 7.097E-02 | 7.230E-02 | 7.080E-01 | 1.030E+00 | 4.850E-01 |
| ZGPAT | 4.744E-01 | 1.696E+00 | 1.289E-04 | 1.839E-01 | 1.850E-01 | 7.910E-01 | 1.120E+00 | 5.600E-01 |
| ZNF142 | 5.790E-01 | 2.491E+00 | 3.904E-04 | 8.177E-02 | 8.310E-02 | 7.220E-01 | 1.040E+00 | 5.000E-01 |
| ZNF146 | 7.058E-01 | 3.888E+00 | 9.439E-06 | 1.709E-01 | 1.720E-01 | 1.280E+00 | 1.830E+00 | 8.970E-01 |
| ZNF280C | 4.514E-01 | 1.059E+00 | 1.904E-03 | 4.188E-01 | 4.190E-01 | 8.740E-01 | 1.210E+00 | 6.300E-01 |
| ZNF282 | 4.213E-01 | 3.356E+00 | 4.058E-03 | 2.552E-03 | 2.840E-03 | 5.960E-01 | 8.370E-01 | 4.250E-01 |
| ZNF316 | 7.167E-01 | 2.853E+00 | 7.275E-05 | 2.142E-01 | 2.150E-01 | 7.820E-01 | 1.150E+00 | 5.300E-01 |
| ZNF318 | 6.445E-01 | 2.314E+00 | 9.708E-05 | 5.513E-01 | 5.510E-01 | 9.060E-01 | 1.250E+00 | 6.540E-01 |
| ZNF326 | 3.469E-01 | 1.875E+00 | 1.145E-02 | 1.254E-01 | 1.270E-01 | 7.490E-01 | 1.090E+00 | 5.170E-01 |
| ZNF511 | 5.541E-01 | 2.623E+00 | 3.361E-03 | 1.868E-02 | 1.950E-02 | 6.770E-01 | 9.390E-01 | 4.880E-01 |
| ZNF518A | 5.032E-01 | 1.832E+00 | 7.624E-03 | 1.736E-01 | 1.750E-01 | 7.940E-01 | 1.110E+00 | 5.690E-01 |
| ZNF593 | 5.604E-01 | 1.608E+00 | 1.459E-03 | 1.600E-01 | 1.620E-01 | 7.860E-01 | 1.100E+00 | 5.620E-01 |
| ZNF598 | 6.695E-01 | 3.574E+00 | 1.319E-04 | 3.126E-01 | 3.130E-01 | 8.460E-01 | 1.170E+00 | 6.110E-01 |
| ZNF622 | 4.918E-01 | 3.696E+00 | 6.592E-03 | 4.083E-01 | 4.080E-01 | 1.150E+00 | 1.610E+00 | 8.250E-01 |
| ZNF623 | 5.212E-01 | 2.129E+00 | 4.302E-04 | 3.403E-01 | 3.410E-01 | 8.530E-01 | 1.180E+00 | 6.160E-01 |
| ZNF672 | 4.033E-01 | 3.332E+00 | 6.208E-03 | 2.505E-02 | 2.610E-02 | 6.720E-01 | 9.540E-01 | 4.730E-01 |
| ZNF69 | 5.411E-01 | 1.155E+00 | 4.878E-03 | 4.040E-01 | 4.050E-01 | 1.180E+00 | 1.730E+00 | 8.010E-01 |
| ZNF697 | 6.702E-01 | 1.134E+00 | 3.947E-06 | 1.658E-02 | 1.770E-02 | 1.650E+00 | 2.500E+00 | 1.090E+00 |
| ZNF7 | 4.483E-01 | 1.333E+00 | 2.475E-06 | 1.587E-01 | 1.600E-01 | 7.550E-01 | 1.120E+00 | 5.100E-01 |
| ZNF703 | 8.698E-01 | 4.994E+00 | 4.418E-02 | 7.890E-02 | 8.010E-02 | 7.310E-01 | 1.040E+00 | 5.150E-01 |
| ZNF706 | 4.232E-01 | 3.410E+00 | 9.640E-03 | 9.596E-02 | 9.740E-02 | 7.230E-01 | 1.060E+00 | 4.920E-01 |
| ZNF761 | 7.569E-01 | 1.683E+00 | 8.387E-07 | 1.431E-01 | 1.440E-01 | 7.650E-01 | 1.100E+00 | 5.340E-01 |
| ZNF783 | 4.255E-01 | 1.278E+00 | 9.015E-04 | 3.335E-02 | 3.430E-02 | 6.860E-01 | 9.720E-01 | 4.830E-01 |
| ZNF787 | 6.408E-01 | 3.624E+00 | 1.570E-03 | 1.889E-02 | 1.970E-02 | 6.730E-01 | 9.390E-01 | 4.820E-01 |
| ZNF792 | 5.666E-01 | 1.304E+00 | 7.704E-05 | 4.674E-01 | 4.680E-01 | 1.140E+00 | 1.630E+00 | 7.990E-01 |
| ZNFX1 | 8.788E-01 | 3.685E+00 | 4.804E-05 | 3.018E-01 | 3.020E-01 | 8.420E-01 | 1.170E+00 | 6.070E-01 |
| ZUFSP | 4.453E-01 | 1.744E+00 | 3.067E-04 | 3.962E-02 | 4.070E-02 | 1.410E+00 | 1.970E+00 | 1.010E+00 |
| RORA | -5.289E-01 | 1.106E+00 | 1.596E-02 | 5.514E-02 | 5.630E-02 | 1.410E+00 | 1.990E+00 | 9.910E-01 |
| THRB | -7.523E-01 | 1.641E+00 | 8.684E-03 | 9.985E-04 | 1.180E-03 | 1.830E+00 | 2.640E+00 | 1.270E+00 |
| ID4 | -1.127E+00 | 3.153E+00 | 1.261E-05 | 4.658E-02 | 4.800E-02 | 1.510E+00 | 2.260E+00 | 1.000E+00 |
| MITF | -6.756E-01 | 1.649E+00 | 8.671E-03 | 5.386E-02 | 5.500E-02 | 1.410E+00 | 2.010E+00 | 9.930E-01 |
| ISL1 | -7.382E-01 | 1.365E+00 | 2.320E-02 | 1.606E-01 | 1.610E-01 | 1.260E+00 | 1.760E+00 | 9.110E-01 |
| MXI1 | -9.319E-01 | 3.308E+00 | 9.045E-05 | 7.937E-02 | 8.070E-02 | 1.440E+00 | 2.160E+00 | 9.570E-01 |
| TSHZ3 | -6.697E-01 | 1.700E+00 | 3.312E-02 | 1.000E-02 | 1.060E-02 | 1.540E+00 | 2.140E+00 | 1.110E+00 |
| FOXF1 | -1.216E+00 | 3.553E+00 | 8.322E-03 | 5.647E-02 | 5.770E-02 | 1.420E+00 | 2.050E+00 | 9.890E-01 |
| FOXN3 | -6.780E-01 | 3.369E+00 | 3.027E-04 | 4.220E-02 | 4.320E-02 | 1.420E+00 | 1.990E+00 | 1.010E+00 |
| POU6F1 | -5.113E-01 | 1.259E+00 | 3.534E-02 | 1.560E-03 | 1.760E-03 | 1.680E+00 | 2.330E+00 | 1.210E+00 |
| MEIS1 | -1.047E+00 | 1.706E+00 | 9.643E-04 | 7.568E-02 | 7.690E-02 | 1.400E+00 | 2.020E+00 | 9.650E-01 |
| ZNF331 | -8.541E-01 | 1.618E+00 | 9.558E-03 | 4.818E-04 | 5.750E-04 | 1.770E+00 | 2.450E+00 | 1.280E+00 |
| KLF15 | -1.463E+00 | 1.708E+00 | 3.681E-05 | 3.719E-01 | 3.730E-01 | 1.190E+00 | 1.720E+00 | 8.150E-01 |
| PBX1 | -8.760E-01 | 2.256E+00 | 8.319E-04 | 1.740E-01 | 1.750E-01 | 1.260E+00 | 1.750E+00 | 9.030E-01 |
| HOXA4 | -1.128E+00 | 1.831E+00 | 8.680E-04 | 2.419E-02 | 2.510E-02 | 1.500E+00 | 2.150E+00 | 1.050E+00 |
| ZBTB4 | -7.463E-01 | 4.279E+00 | 5.507E-03 | 1.112E-01 | 1.120E-01 | 1.310E+00 | 1.830E+00 | 9.390E-01 |
| SMAD9 | -9.575E-01 | 1.715E+00 | 2.259E-03 | 1.572E-01 | 1.580E-01 | 1.280E+00 | 1.790E+00 | 9.100E-01 |
| NR3C2 | -7.418E-01 | 2.015E+00 | 6.943E-03 | 2.137E-01 | 2.140E-01 | 1.230E+00 | 1.700E+00 | 8.870E-01 |
| HOXA2 | -5.670E-01 | 1.019E+00 | 2.368E-02 | 9.431E-02 | 9.570E-02 | 1.390E+00 | 2.040E+00 | 9.440E-01 |
| KLF9 | -1.312E+00 | 4.090E+00 | 3.328E-04 | 2.218E-03 | 2.460E-03 | 1.650E+00 | 2.280E+00 | 1.190E+00 |
| PRDM8 | -7.491E-01 | 1.420E+00 | 2.144E-02 | 6.158E-02 | 6.320E-02 | 1.470E+00 | 2.210E+00 | 9.790E-01 |
| NR3C1 | -8.314E-01 | 2.601E+00 | 2.935E-05 | 1.248E-02 | 1.360E-02 | 1.710E+00 | 2.610E+00 | 1.120E+00 |
| ZBTB16 | -1.347E+00 | 1.414E+00 | 1.565E-04 | 4.170E-03 | 4.510E-03 | 1.600E+00 | 2.220E+00 | 1.160E+00 |
| MEIS2 | -6.185E-01 | 1.649E+00 | 8.931E-03 | 9.063E-02 | 9.220E-02 | 1.370E+00 | 1.970E+00 | 9.500E-01 |
| ZNF518B | -4.271E-01 | 1.093E+00 | 2.528E-02 | 1.377E-01 | 1.390E-01 | 1.300E+00 | 1.840E+00 | 9.190E-01 |
| HAND2 | -2.037E+00 | 1.949E+00 | 3.420E-03 | 9.465E-02 | 9.580E-02 | 1.370E+00 | 2.000E+00 | 9.450E-01 |
| TEF | -8.790E-01 | 2.583E+00 | 4.571E-04 | 9.883E-02 | 9.980E-02 | 1.320E+00 | 1.840E+00 | 9.480E-01 |
| ZSCAN18 | -9.567E-01 | 1.429E+00 | 3.275E-04 | 4.204E-02 | 4.290E-02 | 1.400E+00 | 1.940E+00 | 1.010E+00 |
| FOXF2 | -1.412E+00 | 3.447E+00 | 1.068E-03 | 2.034E-01 | 2.050E-01 | 1.280E+00 | 1.860E+00 | 8.750E-01 |
| HIF3A | -1.355E+00 | 1.457E+00 | 2.152E-03 | 2.201E-02 | 2.320E-02 | 1.600E+00 | 2.390E+00 | 1.070E+00 |
| GATA5 | -1.328E+00 | 1.565E+00 | 1.169E-02 | 2.383E-01 | 2.390E-01 | 1.270E+00 | 1.880E+00 | 8.540E-01 |
| PLAGL1 | -6.043E-01 | 1.593E+00 | 1.534E-02 | 7.537E-04 | 9.210E-04 | 1.890E+00 | 2.760E+00 | 1.300E+00 |
| BARX1 | -1.705E+00 | 3.863E+00 | 3.793E-02 | 1.320E-01 | 1.330E-01 | 1.280E+00 | 1.780E+00 | 9.270E-01 |
| TSHZ1 | -5.923E-01 | 2.585E+00 | 1.201E-02 | 1.955E-01 | 1.970E-01 | 1.260E+00 | 1.800E+00 | 8.860E-01 |
| NKX6-2 | -1.368E+00 | 1.167E+00 | 2.468E-02 | 2.815E-01 | 2.820E-01 | 8.210E-01 | 1.180E+00 | 5.730E-01 |
| SIX2 | -1.309E+00 | 1.960E+00 | 4.381E-02 | 2.315E-01 | 2.320E-01 | 1.220E+00 | 1.710E+00 | 8.780E-01 |
| NFE2L2 | -5.660E-01 | 4.856E+00 | 4.638E-03 | 4.475E-02 | 4.630E-02 | 6.540E-01 | 9.930E-01 | 4.300E-01 |
| DZIP1 | -7.097E-01 | 1.112E+00 | 4.821E-03 | 1.533E-03 | 1.730E-03 | 1.690E+00 | 2.350E+00 | 1.220E+00 |
| SATB1 | -5.864E-01 | 1.881E+00 | 3.527E-02 | 4.456E-02 | 4.600E-02 | 1.490E+00 | 2.210E+00 | 1.010E+00 |
| GLI3 | -6.705E-01 | 1.196E+00 | 6.180E-03 | 1.304E-02 | 1.390E-02 | 1.610E+00 | 2.340E+00 | 1.100E+00 |
| ATOH8 | -7.592E-01 | 1.118E+00 | 9.718E-05 | 2.698E-01 | 2.710E-01 | 1.220E+00 | 1.740E+00 | 8.560E-01 |
| KLF4 | -1.332E+00 | 5.244E+00 | 3.995E-05 | 3.034E-01 | 3.040E-01 | 1.240E+00 | 1.860E+00 | 8.240E-01 |
| ZNF649 | -3.256E-01 | 1.282E+00 | 4.371E-02 | 2.861E-02 | 2.970E-02 | 1.470E+00 | 2.080E+00 | 1.040E+00 |
| HLF | -1.705E+00 | 1.501E+00 | 3.214E-05 | 1.063E-01 | 1.070E-01 | 1.310E+00 | 1.820E+00 | 9.430E-01 |
| MAF | -9.708E-01 | 3.390E+00 | 5.761E-04 | 7.343E-02 | 7.460E-02 | 1.360E+00 | 1.910E+00 | 9.700E-01 |
| ZNF134 | -4.327E-01 | 1.826E+00 | 4.266E-02 | 1.124E-02 | 1.190E-02 | 1.520E+00 | 2.100E+00 | 1.100E+00 |
| C20orf194 | -5.694E-01 | 1.799E+00 | 4.079E-02 | 1.149E-02 | 1.210E-02 | 1.540E+00 | 2.160E+00 | 1.100E+00 |
| CD36 | -1.100E+00 | 1.796E+00 | 9.272E-04 | 7.798E-04 | 9.070E-04 | 1.750E+00 | 2.440E+00 | 1.260E+00 |
| GPR155 | -1.390E+00 | 1.998E+00 | 4.673E-06 | 2.392E-02 | 2.480E-02 | 1.470E+00 | 2.050E+00 | 1.050E+00 |
| LARP6 | -6.352E-01 | 1.493E+00 | 2.790E-02 | 2.643E-03 | 2.910E-03 | 1.640E+00 | 2.280E+00 | 1.190E+00 |
| NFIA | -6.912E-01 | 3.161E+00 | 2.838E-02 | 3.565E-01 | 3.580E-01 | 8.440E-01 | 1.210E+00 | 5.880E-01 |
| NFIX | -6.162E-01 | 4.847E+00 | 4.510E-02 | 7.369E-02 | 7.500E-02 | 7.140E-01 | 1.030E+00 | 4.930E-01 |
| OTOP3 | -1.382E+00 | 1.474E+00 | 3.738E-02 | 1.407E-01 | 1.420E-01 | 7.820E-01 | 1.090E+00 | 5.640E-01 |
| SALL2 | -8.905E-01 | 1.204E+00 | 4.093E-03 | 4.407E-03 | 4.840E-03 | 1.660E+00 | 2.370E+00 | 1.170E+00 |
| SETBP1 | -8.548E-01 | 1.576E+00 | 3.438E-03 | 1.830E-02 | 1.900E-02 | 1.480E+00 | 2.060E+00 | 1.070E+00 |
| SORBS2 | -1.218E+00 | 1.932E+00 | 2.485E-04 | 3.619E-01 | 3.630E-01 | 1.160E+00 | 1.610E+00 | 8.400E-01 |
| SOX15 | -1.503E+00 | 1.914E+00 | 4.176E-03 | 8.465E-02 | 8.550E-02 | 1.330E+00 | 1.850E+00 | 9.610E-01 |
| SOX17 | -6.655E-01 | 1.758E+00 | 3.835E-02 | 4.685E-03 | 5.130E-03 | 1.680E+00 | 2.410E+00 | 1.170E+00 |
| SOX2 | -1.222E+00 | 2.009E+00 | 1.181E-02 | 1.604E-01 | 1.620E-01 | 1.320E+00 | 1.950E+00 | 8.950E-01 |
| SOX21 | -1.889E+00 | 1.895E+00 | 1.214E-03 | 1.231E-01 | 1.240E-01 | 1.310E+00 | 1.840E+00 | 9.290E-01 |
| TCF7L1 | -8.293E-01 | 2.120E+00 | 1.002E-02 | 1.383E-04 | 1.760E-04 | 1.870E+00 | 2.600E+00 | 1.350E+00 |
| TSC22D3 | -1.111E+00 | 4.774E+00 | 1.325E-03 | 2.402E-02 | 2.500E-02 | 1.490E+00 | 2.100E+00 | 1.050E+00 |
| ZFP36 | -1.182E+00 | 7.730E+00 | 3.939E-03 | 1.082E-02 | 1.140E-02 | 1.550E+00 | 2.180E+00 | 1.100E+00 |
| ZNF595 | -4.406E-01 | 1.298E+00 | 4.531E-02 | 3.394E-02 | 3.520E-02 | 1.520E+00 | 2.240E+00 | 1.030E+00 |

**Supporting table S2. The evaluation of prognosis effects of TME cells in TCGA-STAD cohort.**

|  | Log.rank.p | Uni.cox.p | HR | HR.upper | HR.low |
| --- | --- | --- | --- | --- | --- |
| Fibroblasts | 0.010 | 0.011 | 1.530 | 2.130 | 1.100 |
| Stroma Score | 0.055 | 0.057 | 1.390 | 1.960 | 0.991 |
| B.cells.naive | 0.109 | 0.110 | 1.310 | 1.810 | 0.942 |
| T.cells.CD4.naive | 0.576 | 0.576 | 1.290 | 3.150 | 0.528 |
| Endothelial cells | 0.159 | 0.159 | 1.260 | 1.750 | 0.912 |
| Immune Score | 0.174 | 0.175 | 1.260 | 1.750 | 0.903 |
| Macrophages.M2 | 0.174 | 0.174 | 1.250 | 1.740 | 0.905 |
| Monocytes | 0.242 | 0.242 | 1.230 | 1.740 | 0.869 |
| Microenvironment Score | 0.243 | 0.244 | 1.210 | 1.690 | 0.876 |
| Dendritic.cells.resting | 0.400 | 0.401 | 1.160 | 1.640 | 0.821 |
| T.cells.CD4.memory.resting | 0.404 | 0.403 | 1.150 | 1.590 | 0.829 |
| Macrophages.M1 | 0.515 | 0.515 | 1.110 | 1.540 | 0.805 |
| Neutrophils | 0.584 | 0.583 | 1.110 | 1.580 | 0.772 |
| Macrophages.M0 | 0.755 | 0.755 | 1.050 | 1.460 | 0.759 |
| T.cells.gamma.delta | 0.883 | 0.884 | 1.040 | 1.680 | 0.640 |
| Mast.cells.resting | 0.903 | 0.903 | 1.020 | 1.430 | 0.730 |
| Eosinophils | 0.949 | 0.949 | 1.020 | 1.800 | 0.576 |
| Mast.cells.activated | 0.941 | 0.941 | 1.010 | 1.440 | 0.711 |
| NK.cells.activated | 0.977 | 0.976 | 0.995 | 1.400 | 0.709 |
| B.cells.memory | 0.884 | 0.884 | 0.968 | 1.500 | 0.624 |
| T.cells.CD8 | 0.666 | 0.665 | 0.930 | 1.290 | 0.668 |
| Dendritic.cells.activated | 0.675 | 0.675 | 0.927 | 1.320 | 0.649 |
| Epithelial cells | 0.534 | 0.534 | 0.898 | 1.260 | 0.638 |
| NK.cells.resting | 0.403 | 0.403 | 0.860 | 1.230 | 0.603 |
| Plasma.cells | 0.325 | 0.325 | 0.842 | 1.190 | 0.598 |
| T.cells.regulatory.Tregs. | 0.090 | 0.092 | 0.755 | 1.050 | 0.545 |
| T.cells.follicular.helper | 0.084 | 0.085 | 0.742 | 1.040 | 0.529 |
| T.cells.CD4.memory.activated | 0.009 | 0.010 | 0.621 | 0.892 | 0.433 |

| **Supporting table S3. Primers, sgRNA sequences and siRNA sequences.** | |
| --- | --- |
| qPCR primers | |
| HEYL-qF | CCATCGACGTGGGCCAAGA |
| HEYL -qR | TCTCGACGCCGTTTCTCTATG |
| CDH11-qF | CATCCCAAGAGAGGTCTGCG |
| CDH11-qR | TACGTGGTAGGCACAGGAGA |
| CDH11-pro-qF | GGTAGCGAGTCGTCAGTGAG |
| CDH11-pro-qR | GAGTGTGCGTTTCTACGGGA |
| ACTB-qF | TTGTTACAGGAAGTCCCTTGCC |
| ACTB-qR | ATGCTATCACCTCCCCTGTGTG |
|  |  |
| HEYL deletion sequences | |
| sgHEYL-F1 | CCTTTCTGAATTGCGACGCT |
| sgHEYL-F2 | GGGTCCGTCGGACTCCCCGT |
| sgHEYL-F3 | GTTCGCCATGAAGCGACCCA |
|  |  |
| siRNA sequences | |
| siHEYL-1 | AGGAAGTAGGAAGAGTGAAAGCTAA |
| siHEYL-2 | CAGCCTGGCTCAACATCACTGTCTT |

| **Supporting table S4. Antibodies used in this study.** | | | |
| --- | --- | --- | --- |
| Name | Company | Catalog Number | Use |
| HEYL | Proteintech | 15679-1-AP | WB, ChIP |
| CDH11 | Thermo Fisher | 32-1700 | WB |
| ACTB | Sigma-Aldrich | A2228 | WB |
