# Supplementary material for: Charactering tumor microenvironment reveals stromal‐related transcription factors promote tumor carcinogenesis in gastric cancer
Source: Cancer Med. 2020 May 28;9(14):5247–57. doi: 10.1002/cam4.3133 (PMC7367614; doi:10.1002/cam4.3133)
Supplement: Supplementary file 2 — Supplementary Material [file CAM4-9-5247-s002.docx]

**Supporting Materials**

**Charactering Tumor Microenvironment Reveals Stromal Related Transcription Factors Promote Tumor Carcinogenesis in Gastric Cancer**

**Estimation of infiltrating cells in TME**

To enumerate the infiltrating fractions of immune cells in the TCGA-STAD samples, we implemented the CIBERSORT algorithm and the LM22 immune cell gene set signature, which provide the sensitive and specific investigation of full 22 human immune cell types. The CIBERSORT is a defined as a deconvolution strategy which utilizes the candidate gene expression profile of all immune cells (a signature with 547 genes) regarded a minimal exhibition for 22 types of immune cell and, according to these values, provides cell type compositions in expression profile from bulk tumor samples with heterogenetic component types by support vector regression. The expression profiles of RNA-seq results were constructed using standard annotation files, and these datasets were presented to the CIBERSORT web set (http://cibersort.stanford.edu/), with the method performs using the LM22 gene set and 1,000 iterations. Tumor purity was evaluated by ESTIMATE method. The proportions of endothelial cells, epithelial cells, fibroblasts, immune score and microenvironment score were estimated by applying the xCell method, the stromal cells component were represented by stromal score, which allows for robust quantification of the absolute abundance of TME cells populations in heterogeneous tissues from TCGA-STAD transcriptomic data.

**Datasets download and preprocessing**

Data from The Cancer Genome Atlas (TCGA) were downloaded from the UCSC Xena browser (GDC hub, https://xena.ucsc.edu/), as detailed in the supplementary methods. For TCGA-STAD dataset, RNA-sequencing profile were transformed into Fragments Per Kilobase Million (FPKM) values. The clinical data and sample information for TCGA-STAD cohort were obtained from the Genomic Data Commons (https://portal.gdc.cancer.gov/) using the R package *TCGAbiolinks*.

**Functional and pathway enrichment analysis**

Gene-annotation enrichment analysis using the *clusterProfiler* R package was performed on different expressed genes. Gene Ontology (GO) terms were identified with a false discovery rate (FDR) of less than 0.05. We also identified pathways that were up-regulated in TME-clusters A for TCGA-STAD and RNA-seq results by running a gene set enrichment analysis (GSEA) of expression profile of all DEGs. Cancer hallmark gene sets were downloaded from the MSigDB database of Broad Institute. Enrichment *P* values were based on 10,000 permutations and subsequently adjusted for multiple testing using the Benjamini-Hochberg procedure to control the FDR. Differentially regulated genes were assessed for KEGG pathway enrichment using DAVID platform (<https://david.ncifcrf.gov/>).

**Cell lines and cell culture**

The human gastric cancer cell lines HGC-27 and AGS were obtained from the Shanghai Cell Bank Type Culture Collection Committee (CBTCCC, Shanghai, China). The human embryonic kidney 293T cells were purchased from American Type Culture Collection (ATCC, Manassas, Virginia, USA). HGC-27 and AGS cells were cultured in RPMI1640 (Thermo Fisher Scientific, Waltham, MA, USA) and HEK-293T cells in DMEM (Gibco, Grand Island, NY, USA) supplemented with 10% fetal bovine serum (Gibco), 100 µg/ml penicillin (Gibco) and 100 µg/ml streptomycin (Gibco) at 37°C and 5% CO_2_ . Cells were added with Mycoplasma-OUT (Genechem, Shanghai, China) for 2 week and examined with Mycoplasma Test Kit (Genechem) to ensure the free of mycoplasma contamination.

**RNA extraction, reverse transcription, RT-qPCR analysis**

Total RNAs were extracted from cells and tissues using TRIzol reagent (Invitrogen, Carlsbad, CA, USA). cDNA was synthesized using PrimeScript RT Reagent Kit (TaKaRa, Shiga, Japan). RT-qPCR was done using SYBR Premix *Ex Taq II* (TaKaRa). The relative expression of RNA was determined QuantStudio 7 Flex sequence detection system (Thermo Fisher Scientific, Waltham, MA, USA). The specific genes primers are listed in Table S3.

**RNA interference**

Small interfering RNA (siRNA) oligonucleotides targeting HEYL were designed and synthesized by RiboBio (Guangzhou, China). Gastric cancer cells HGC-27 and AGS were transfected with siRNAs using the Lipofectamine® RNAiMAX reagent (Invitrogen) at a final concentration of 50 nM. After transfection for 48 hours, these cells were used for RNA extraction, immunoblotting, proliferation and migration assays. The sequences for HEYL siRNAs used are listed in Table S3.

**Lentivirus production and transduction**

The packaging plasmid psPAX2 and the VSV-G envelope plasmid pMD2.G (gifts from Dr. Didier Trono) combined with HEYL or CDH11 were transfected into HEK293T cells using Lipofectamine® 2000 (Invitrogen). Lentiviral particles were harvested at 48 h after transfection. The gastric cancer cells were infected with recombinant lentivirus and 6 µg/mL polybrene (Sigma-Aldrich, Missouri, USA).

**Colony formation and migration assays**

For the colony formation assay, 1.5 × 10^3^ cells were seeded in a hole of 6-well plate and incubated at 37°C for nearly 10 days. The colonies stained with 100% methanol and dye solution containing 0.5% crystal violet (Sigma-Aldrich) followed with counted. Cell migration assays were performed in a 24-well plate with 8-µm pore size chamber inserts (BD Biosciences, Franklin Lakes, NJ). 5×10^4^ cells were suspended in the upper chamber with 200 µL of Dulbecco’s modified Eagle’s medium (DMEM) without fetal bovine serum (FBS) per well; 800 µL of DMEM with 10% FBS was added to the lower chamber. The chambers were fixed and stained with 100% methanol and dye solution containing 0.5% crystal violet after 20 hours of incubation at 37°C, followed by imaging under inverted microscope (Olympus, Tokyo, Japan).

**Xenograft in nude mice**

HEYL knockdown AGS cells used by CRISPR/Cas9 system and control cells were digested and re-suspended in RPMI-1640 without FBS. Twelve mice (male BALB/c-nu/nu, 6 weeks old) were randomly divided into two groups. Each group of mice were subcutaneously injected in the lower back with 2 × 10^6^ cells in 200 µL of RPMI-1640 without FBS. The mice were sacrificed, and the tumors were dissected and weighed at approximately 4 weeks after injection. The mouse experiments were conducted by the Guide for the Care and Use of Laboratory Animals of Fudan University.

**Chromatin immunoprecipitation (ChIP) assay**

ChIP assay was conducted by Simple ChIP Plus Sonication Chromatin IP Kit (Cell Signaling Technology, Boston, USA). In brief, gastric cancer cells were cross-linked followed by sonicated with Bioruptor UCD-200 (Diagenode, Liege, Belgium). Chromatin was immunoprecipitated with antibodies against HEYL on Dynabeads ® Protein G (Thermo Fisher Scientific) and digested with proteinase K and RNase A. DNA was extracted with MinElute Reaction Cleanup Kit (Qiagen, Hilden, Germany). ChIP-qPCR was performed with QuantStudio 7 Flex sequence detection system using SYBR Premix Ex Taq II (Takara). Primers were listed in Table S3, and antibodies were provided in Table S4.


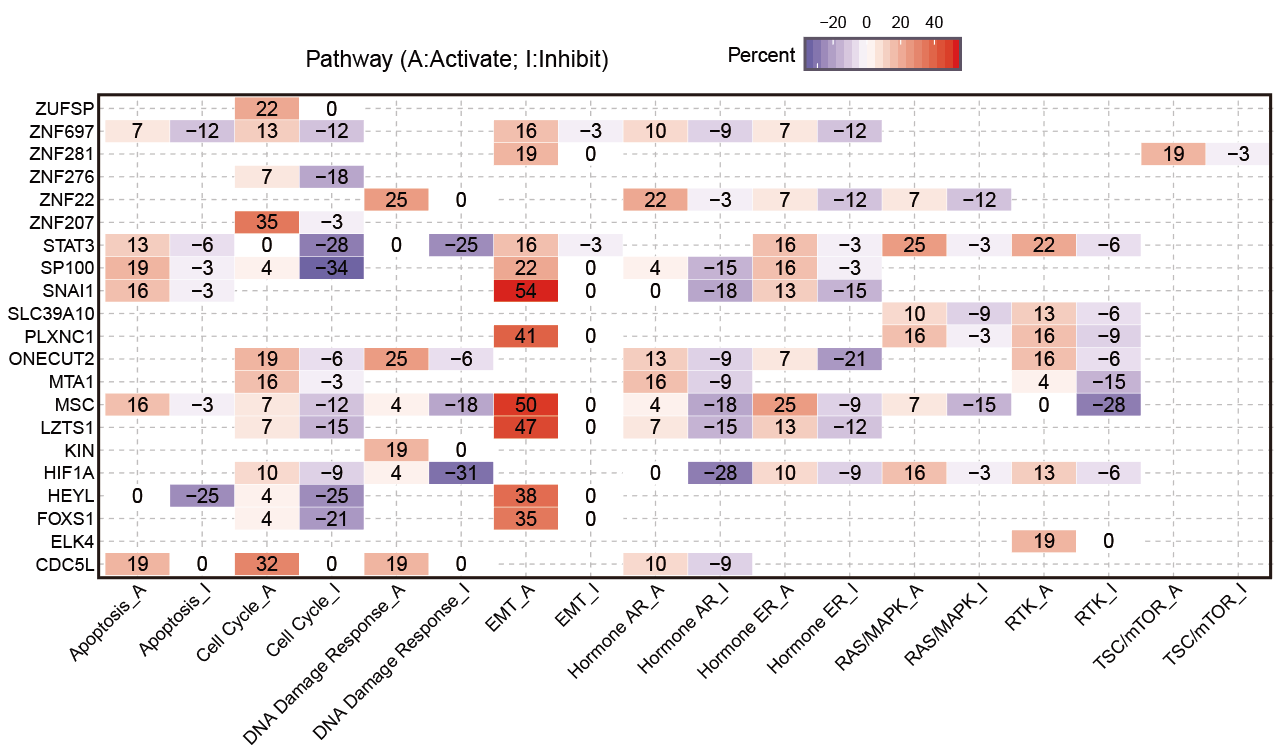


**Fig. S1.** The annotation of biological functions of risk TFs by GSCALite (Gene Set Cancer Analysis). The GSCALite output results of gene set annotation demonstrated by percentage ratio and the HEYL was highly correlated to EMT activation.


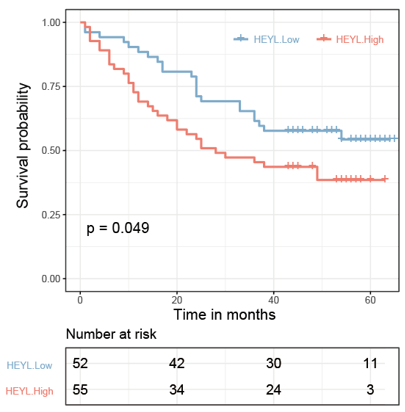


**Fig. S2**. The disease-free survival plot of HEYL generated by Kaplan-Meier Survival analysis in our internal cohort.


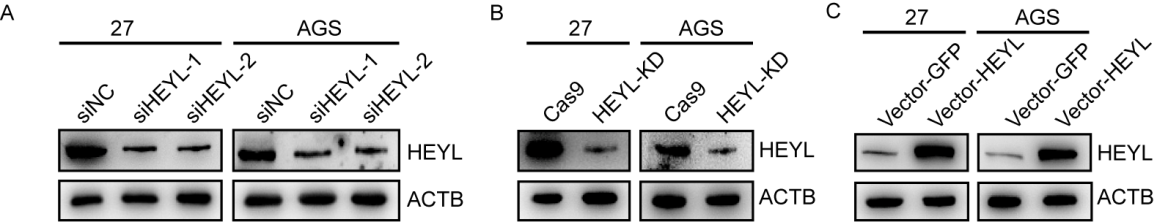


**Fig. S3.** Immunoblotting for HEYL protein levels in gastric cancer cells. (A) Immunoblotting for HEYL protein levels in HGC-27 and AGS cells transfected with HEYL siRNAs or negative control (NC) siRNA. (B) Immunoblotting for HEYL protein levels in HGC-27 and AGS cells infected with HEYL knockdown sgRNA or control sgRNA lentivirus. (C) Immunoblotting for HEYL protein levels in HGC-27 and AGS cells infected with HEYL or GFP overexpression lentivirus. β-actin served as loading control.


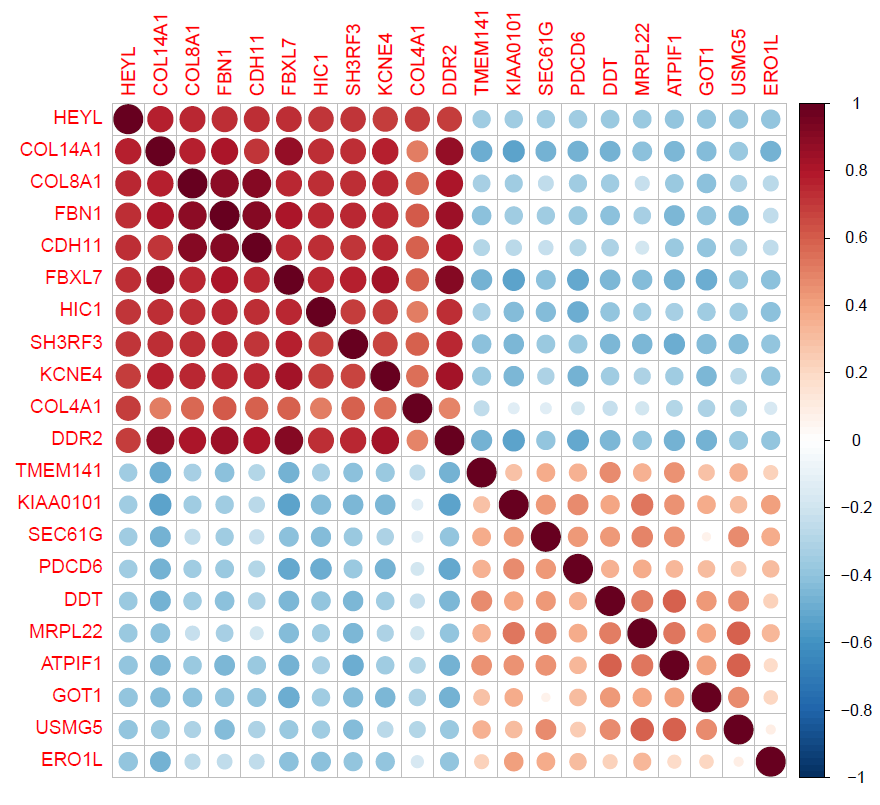


**Fig. S4.** The expressional correlation between HEYL and regulated genes. Top ten up- and down-regulated genes with HEYL mRNA levels developed by HEYL-KD RNA-seq results. Note that the mRNA levels of COL14A1, COL8A1, FBN1 and CDH11 are positively correlated with HEYL mRNA levels.


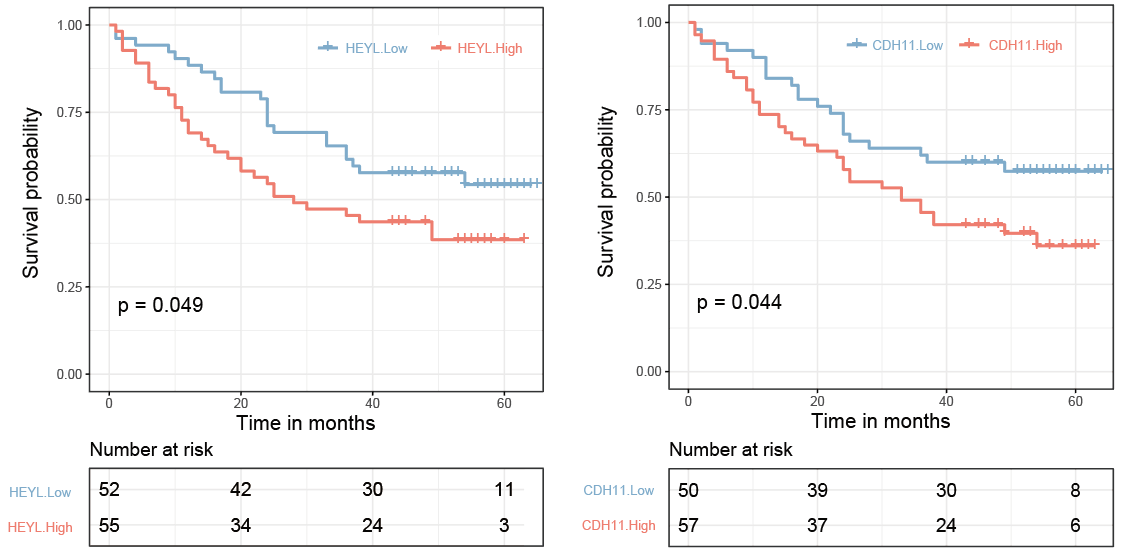


**Fig. S5**. The disease-free survival plot of CDH11 generated by Kaplan-Meier Survival analysis in our internal cohort.
